# Part I: consensus statements and expert recommendations for *HER2*-negative early breast cancer in the Asia-Pacific region: diagnosis and risk assessment

**DOI:** 10.3389/fonc.2025.1507836

**Published:** 2025-06-23

**Authors:** Soo Chin Lee, Yeon Hee Park, Christian F. Singer, Judith Balmaña, Rebecca Alexandra Dent, Veronique Kiak-Mien Tan, Nadia Ayu Mulansari, Mastura Md Yusof, Frances Victoria F. Que, Yen-Shen Lu, Napa Parinyanitikul, Cam Phuong Pham, Nur Aishah Taib, Sun-Young Kong, Yoland Antill, Hee Jeong Kim

**Affiliations:** ^1^ Department of Haematology-Oncology, National University Cancer Institute, Singapore, Singapore; ^2^ Division of Haematology-Oncology, Department of Medicine, Samsung Medical Center, Sungkyunkwan University School of Medicine, Seoul, Republic of Korea; ^3^ Department of Obstetrics and Gynaecology, Comprehensive Cancer Center, Medical University of Vienna, Vienna, Austria; ^4^ Department of Medical Oncology, University Hospital Campus Vall Hebron, Barcelona, Spain; ^5^ Division of Medical Oncology, National Cancer Centre Singapore, Singapore, Singapore; ^6^ Division of Surgery and Surgical Oncology, Department of Breast Surgery, National Cancer Centre Singapore, Singapore, Singapore; ^7^ Haematology-Medical Oncology Division, Internal Medicine Department, Cipto Mangunkusumo National General Hospital/Universitas Indonesia, Jakarta, Indonesia; ^8^ Picaso Cancer Centre, Hospital Picaso, Petaling Jaya, Selangor, Malaysia; ^9^ Department of Internal Medicine and Oncology, St Luke’s Medical Center, Quezon City and Global City, Metro Manila, Philippines; ^10^ Department of Oncology, National Taiwan University Hospital, Taipei, Taiwan; ^11^ Medical Oncology Unit, King Chulalongkorn Memorial Hospital, Bangkok, Thailand; ^12^ Nuclear Medicine and Oncology Center, Bach Mai Hospital, Hanoi, Vietnam; ^13^ Department of Surgery, Faculty of Medicine, University Malaya, UM Cancer Research Institute, Kuala Lumpur, Malaysia; ^14^ Department of Laboratory Medicine and Genetic Counselling Clinic, National Cancer Center, Goyang, Republic of Korea; ^15^ Familial Cancer Centre, Royal Melbourne Hospital, Melbourne, VIC, Australia; ^16^ Faculty of Medicine and Health Sciences, Monash University, Melbourne, VIC, Australia; ^17^ Division of Breast Surgery, Department of Surgery, Asan Medical Center, University of Ulsan College of Medicine, Seoul, Republic of Korea

**Keywords:** *BRCA* germline pathogenic variants, consensus, early breast cancer, *HER2*, HR+, triple-negative breast cancer, recurrence

## Abstract

**Introduction:**

In the Asia-Pacific region, there is increasing contention on the practical challenges involved in managing human epidermal growth factor receptor 2 (HER2)-negative early breast cancer (eBC). This modified Delphi consensus explores gaps in genetic counselling (GC) and genetic testing (GT), and clinical risk assessment for *HER2*-negative eBC.

**Methods:**

An expert panel of 16 Asia-Pacific medical oncologists, geneticists, and breast cancer surgeons arrived at 33 statements. The level of statement consensus was considered high at ≥75%. A survey of 134 healthcare practitioners (HCPs) (breast cancer surgeons, geneticists, oncologists, molecular biologists/pathologists) explored the real-world practices in this region.

**Results:**

A consensus was reached for 88% of the statements (29/33) and aligned with international guidelines. Experts reached 100% consensus on offering pretest GC, obtaining consent before GT, considering first diagnosis of breast cancer (BC) as ideal time for GT, offering reflex testing for patients with likely/pathogenic germline *BRCA* variant, and considering patients with germline *BRCA* mutant early triple-negative breast cancer (TNBC) patients who do not achieve pathological complete response after neoadjuvant treatment to be at high risk of recurrence. Over 90% of experts supported germline GT for *BRCA* for TNBC patients irrespective of age at diagnosis or family history and prioritised tumour size and nodal status as prognostic factors for cancer recurrence. Experts reached 80%-90% consensus for using genetic risk assessment tools in low/under-resourced healthcare systems and considering patients with likely/pathogenic variants in *BRCA* for risk reduction surgery. Significant gaps existed between real-world practices and recommendations, particularly in offering pretest GC to patients with suspected hereditary BC and to blood relatives of patients with *BRCA* germline pathogenic variant BC, ideal time for GT, considering GT for early TNBC patients irrespective of age, offering post-test GC for positive results, utilising risk assessment tools, and streamlining GC through non-geneticist HCPs.

**Conclusion:**

GT and pretest GC should be mainstreamed at the first diagnosis of BC. Risk assessment for disease recurrence should be performed at diagnosis and post-surgery for *HER2*-negative eBC patients. These recommendations would help standardise GC and improve GT access for clinical decisions.

## 1 Introduction

Breast cancer (BC) is a significant public health concern and the prevalent reason for cancer-related death among Asian women (1). In, 2020, 2·3 million new BC cases, accounting for 11·7% of all cancers, were diagnosed worldwide among women, surpassing lung cancer (11·4%), with almost half (45·4%), or around 1·0 million cases, being diagnosed in Asia (1–4). BC is the leading cause of cancer-related mortality among women, accounting for 6·9% of the cancer-related deaths across the world.^4^ Age-incidence curves of BC in Asian countries resemble those in the United States (US). However, the incident rates of BC in Asian cohorts are converging and even surpassing US levels, highlighting the urgent need for effective prevention and treatment strategies (5).

Many factors are linked to women’s increased susceptibility to BC in Asian countries. These include vitamin D deficiency and a higher intake of total n-6 polyunsaturated fatty acids, salt, sugar, meat, saturated fat, and oils (6). Other modifiable factors, such as urbanisation, higher body mass index, and low physical activity, are also linked to the increased incidence of BC. Hormonal and reproductive risk factors, such as nulliparity, advanced age at first pregnancy, longer oestrogen exposure, early menarche, and late menopause, predispose women to the risk of developing BC. Similar sets of hormonal, acquired, and intrinsic risk factors for BC have been recognised in both East Asian and Western women. However, the degree of exposure to each factor may differ based on a woman’s ethnicity, cultural background, and place of residence. These disparities in exposure contribute to the lower incidence of BC in East Asian women compared to their Western counterparts (3, 7).

Screening and early diagnosis are vital for successful BC treatment, as the best survival rates are observed in patients with early-stage BC. However, late presentation of the disease, stage III or IV at diagnosis, is typical in >50% of the patients in Asia-Pacific’s low- and middle-income countries (8). The stage at which BC is diagnosed largely depends on social and cultural factors, access to healthcare, and the country’s economic status (8, 9).

According to the Cancer Risk Estimates Related to Susceptibility (CARRIERS) Consortium, 2021, the prevalence of pathogenic variants of *BRCA* among women with BC was estimated to be 1·3% (associated with *BRCA2*) and 0·8% (associated with *BRCA1*) (10). The prevalence of pathogenic variants of *BRCA*1/*2* among women with BC is not significantly different across South Asian countries (11). Furthermore, the risk of BC associated with the *BRCA1/2* gene is also comparable between Asian and European countries, as reported by population-based studies (12). It is estimated that <5% of *BRCA1* and *BRCA2* carriers among women with BC are identified in Asia due to major gaps in the availability of cancer-specialised genetic counselling (GC) and access to funded genetic testing (GT) in this region. However, if GC/GT were more available, the percentage of carriers with *BRCA1* and *BRCA2* would be higher (10, 13).

The potential barriers to GT and GC in the Asia-Pacific region include a shortage of trained genetic counsellors, lack of expertise among physicians, lack of defining criteria for identifying high-risk patients, lack of perceived benefits of GC, lack of perceived risk of having a pathogenic or likely pathogenic variant, cost of testing, and fear of insurance discrimination. Various cultural issues, such as family values, religious principles, beliefs and practices influence GT and GC in low- and middle-income countries (14–18). Furthermore, there is a lack of consensus on genes that should be tested in different clinical scenarios (19).

Regardless of advancements made in the management of *BRCA* germline pathogenic variant early breast cancer (eBC) worldwide, healthcare practitioners (HCPs) still encounter numerous difficult clinical situations in the real world, for which evidence is lacking. Expert opinion is crucial for directing the management of these contentious situations. The challenge of effectively identifying and diagnosing patients with an inherited higher lifetime risk of BC remains unaddressed (19). Additionally, there is a lack of consensus on the guidelines for the identification of *BRCA* germline pathogenic variant carriers in the Asia-Pacific region. Moreover, applying Western guidelines without considering differences in the Asia-Pacific population’s BC natural history, disease biology, epidemiology, and pharmacogenomics, as well as individual countries’ cultural and social backgrounds and resource availability, can lead to suboptimal outcomes (20). Having region-specific guidelines will aid in catering to the region’s distinct demographic profiles, cultural practices, and genetic predispositions, ensuring that recommendations are relevant and effective for local populations. It will also aid in accommodating the diverse healthcare infrastructures across Asia-Pacific by providing practical strategies that align with varying resource availability and are culturally sensitive, thereby improving patient acceptance and compliance, harmonising healthcare practices with regional norms and beliefs. This paper aims to develop tailored practical recommendations for the management of eBC with pathogenic germline *BRCA* variant in the Asia-Pacific region.

### 1.1 Objectives

These consensus statements and expert recommendations aim to aid HCPs in decisions regarding (a) GC and testing in human epidermal growth factor receptor 2 (*HER2*)*-*negative eBC patients for surgical and therapeutic approaches and (b) clinical risk assessment for disease recurrence in *HER2-*negative eBC.

## 2 Methods

**Figure 1:**
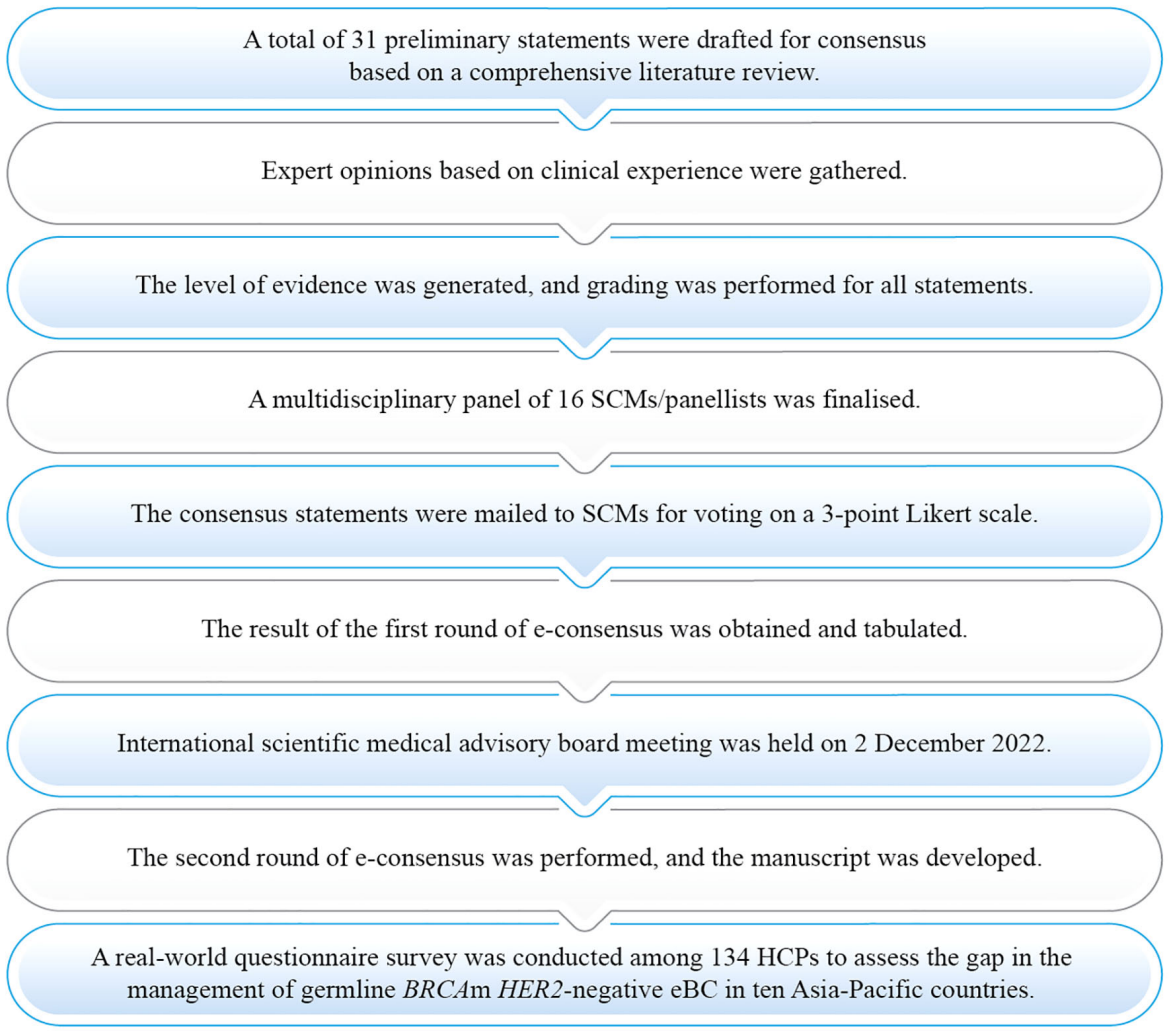
Process of consensus development. eBC, Early breast cancer; HCP, Healthcare practitioner; *HER*2, Human epidermal growth factor receptor 2; m, Mutation; SCM, Steering committee member.

A modified Delphi technique was undertaken with two online surveys and one scientific advisory board meeting. The steps employed in formulating this consensus are presented in **Figure 1**. A set of 31 preliminary statements were drafted by core group members. A multidisciplinary panel of 16 medical oncologists, geneticists, and BC surgeons from the Asia-Pacific region formed the core group of steering committee members (SCMs). The experts were selected as per convenience and were well-renowned in the Asia-Pacific region for the management of BC. The criteria for selecting the experts were: (a) Experts having more than ten years of experience in the same field and (b) having published articles in peer-reviewed journals (**Supplementary Table S1**). There were more medical oncologists and breast surgeons than geneticists in the multidisciplinary panel because the consensus statements were mostly related to treatment practice. The proportion of geneticists was consistent with the proportion of consensus statements that required a deeper understanding of genetics. The consensus statements were created through an extensive literature review in PubMed, EMBASE, and Cochrane databases. Search terms were developed using a combination of medical subject headings (MeSH^®^), Embase’s thesaurus (EMTREE^®^) terms, and free-text keywords related to the objectives (**Supplementary Table S2**). Citations were screened based on title and abstract for relevance. The reference list of each identified article was reviewed for other potentially relevant papers. The literature review included original articles, systematic reviews, and national and international guidelines. A few of the consensus statements were developed based on routinely encountered clinical scenarios suggested by clinicians. Publications from the Asia-Pacific region were considered to identify the gaps in the literature.

The levels of evidence and grades of recommendation were generated for each statement using a modified Grading of Recommendations, Assessment, Development, and Evaluations (GRADE) methodology (**Table 1**) (21). The level of evidence supporting each recommendation statement was categorised from 1 to 5. It was given to studies per their methodological design quality, validity, and suitability for use in patient care (**Table 2**) (22). As a part of the first round of the survey, 31 statements were mailed to the SCMs using the online survey method on a 3-point Likert scale (agree, disagree, and abstain). The results of the first round of e-consensus were tabulated. A statement was considered to have reached a high, moderate, or low consensus when ≥75%, 55%–74%, or <55% of the participants, respectively, agreed or disagreed with the statement. The panellists were asked to vote for the statement as follows: (i) yes, if they agreed; (ii) no, if they disagreed; and (iii) abstain, if they were undecided about the response to that statement or did not have the appropriate expertise to respond to the statement. For each round of modified Delphi techniques, the participant count remained constant, with 16 SCMs involved. However, to determine the level of consensus, experts who abstained from voting due to a perceived lack of expertise or conflict of interest were removed from the denominator for the final calculation of the rate of agreement or disagreement. Hence, statements with denominators less than 16 do not include responses from experts who cited a lack of expertise as their reason for abstaining. The details of the response for each statement are presented in the consensus statement section of this paper.

**Table 1:** Grading of recommendations using the Oxford level of evidence (21).

Code	Quality	Definition
A	High	Further research is very unlikely to change our confidence in the estimate of effect. • Several high‐quality studies with consistent results • In special cases – one large, high‐quality multicentre trial
B	Moderate	Further research is likely to have an important impact on our confidence in the estimate of effect and may change the estimate. • One high‐quality study • Several studies with some limitations
C	Low	Further research is very likely to have an important impact on our confidence in the estimate of effect and is likely to change the estimate. • One or more studies with severe limitations
D	Very low	Any estimate of the effect is very uncertain. • Expert opinion • No direct research evidence • One or more studies with very severe limitations

Grading of Recommendations Assessment, Development, and Evaluation (GRADE) Working Group, 2007 (modified by the Evidence-Based Medicine Guidelines Editorial Team).

**Table 2:** Level of evidence using the Oxford level of evidence (22).

Level of evidence	Therapy/prevention, aetiology/harm	Prognosis
1a	Systematic review (with homogeneity) of RCTs	Systematic review (with homogeneity) of inception cohort studies; clinical decision rule validated in different populations
1b	Individual RCT (with narrow CI)	Individual inception cohort study with >80% follow-up; clinical decision rule validated in a single population
1c	All or none	All or none case series
2a	Systematic review (with homogeneity) of cohort studies	Systematic review (with homogeneity) of either retrospective cohort studies or untreated control groups in RCTs.
2b	Individual cohort study (including low**-**quality RCTs, <80% follow-up)	Retrospective cohort study or follow-up of untreated control patients in an RCT; derivation of clinical decision rule or validated on split-sample only
2c	“Outcomes” research and ecological studies	“Outcomes” research
3a	Systematic review (with homogeneity) of case-control studies	
3b	Individual case-control study	
4	Case series (and poor-quality cohort and case-control studies)	Case series (and poor-quality prognostic cohort studies)
5	Expert opinion without an explicit critical appraisal, or based on physiology, bench research, or “first principles”	Expert opinion without an explicit critical appraisal, or based on physiology, bench research, or “first principles”

CI, Confidence interval; RCT, Randomised controlled trial.

Grading of Recommendations Assessment, Development, and Evaluation (GRADE) Working Group, 2007 (modified by the Evidence-Based Medicine Guidelines Editorial Team).

Round one of the e-consensus survey was followed by an advisory board meeting to discuss the statements that had not reached a consensus. During the meeting, the reason for disagreement was highlighted, and accordingly, the statements were modified in an attempt to reach a consensus. Discussions and comments of the SCMs were documented. Based on the discussion of the SCMs, revised statements were included for a second round of e-survey. The outcomes of all three modified Delphi rounds were merged to report the results on the consensus.

A questionnaire-based survey containing 23 questions derived from the preliminary consensus statements was conducted in parallel to assess the current treatment decisions based on the participants’ clinical experience of the management of *BRCA* germline pathogenic variants in *HER2*-negative eBC in the Asia-Pacific region. It should be noted that the survey questionnaire was developed in consultation with the panel members and rolled out to a broad number of breast cancer experts (n=380) in the Asia-Pacific region. A descriptive approach was used for sampling with no objective of proving or disproving any theories. The survey was conducted with the sole objective of highlighting the unmet needs and gaps in real-world practices to complement the expert recommendations, and not to refine or modify the final recommendations. A total of 134 HCPs from the Asia-Pacific region participated in the survey. The questionnaire was distributed among HCPs practising in the Asia-Pacific region only (the sample for the questionnaire survey was different than that of the Delphi consensus). The demographic and professional details of the HCPs are presented in **Supplementary Figures S1** and **S2**. Periodic reminders were sent to the HCPs for completing the survey to maximise the response rate. No institutional ethical committee approval was taken for the survey, as all the questions in the survey were focussed on the clinical experts’ practices, trends, and opinions on breast cancer. No patient data in any capacity was used in the development of this manuscript except for data already published in various journals.

## 3 Results

**Figure 2:**
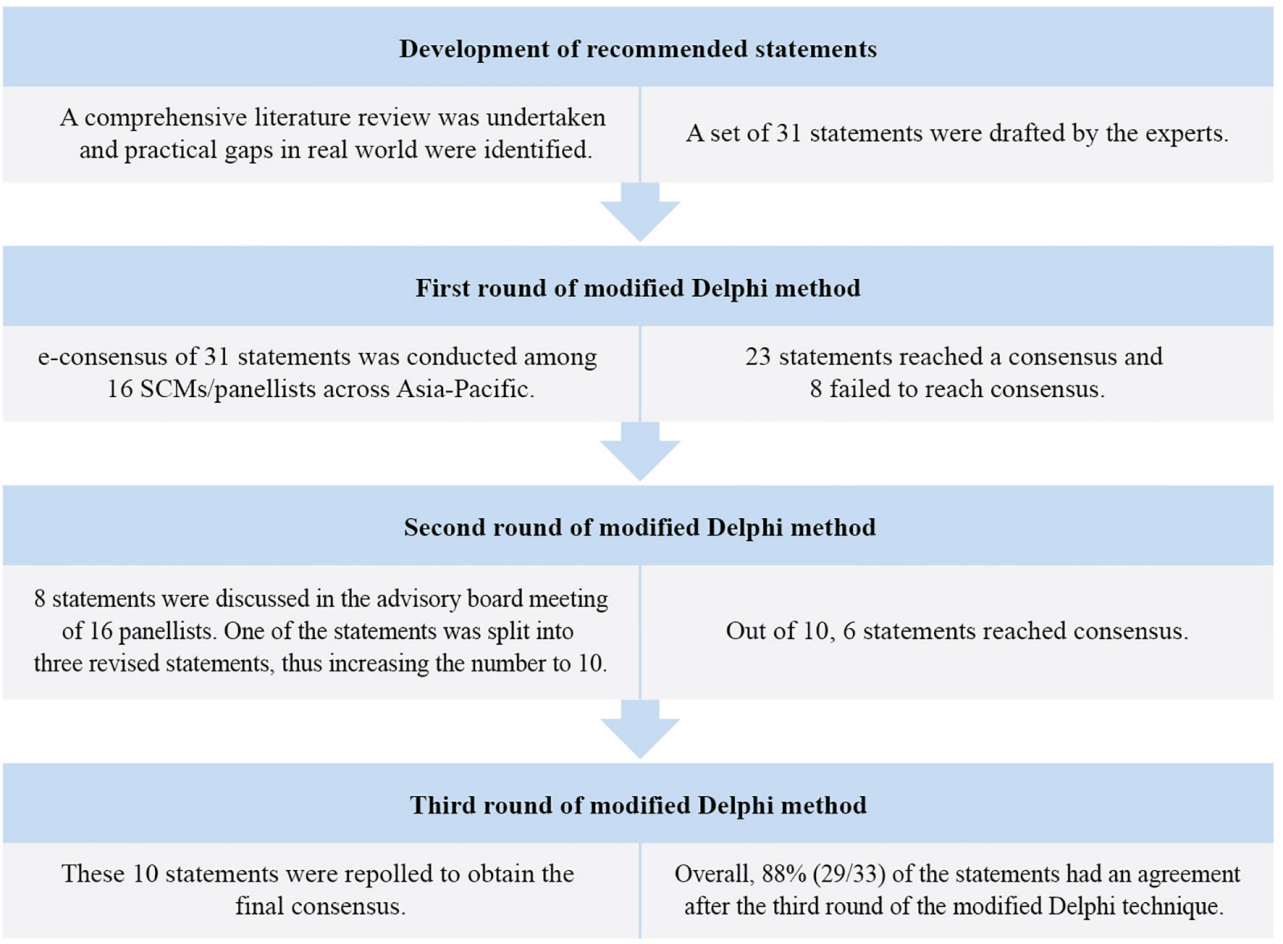
Modified Delphi method for the development of consensus.

Of the 31 statements in the first round of the e-consensus survey, 23 reached a high consensus, whereas eight did not reach a consensus. The eight statements that did not reach initial consensus were discussed and revised during the scientific advisory board meeting. One of the statements was split into three revised statements, increasing the number of statements from 31 to 33. Overall, an agreement was reached for 88% (29/33) of the statements after the third round of the modified Delphi technique (**Figure 2**).

The agreement that is reached, levels of evidence, and grades of recommendation of the consensus statements are provided in **Tables 3**–**5**. **Supplementary Table S3** detail each consensus statement’s agreement, disagreement, and abstain voting percentage. In addition, areas of research for future prospective clinical trials were identified. The statements were graded for quality of evidence using the Oxford Level of Evidence. About 17 statements (51·5%) were of high quality and 16 statements (48·5%) were of very low quality. The level of consensus among the SCMs was high for 28 statements (84·8%), moderate for two statements (6·1%), and low for three statements (9·1%). Results are summarised under the domains outlined in **Tables 3**–**5**.

**Table 3:** Level of consensus on the pre- and post-test GC in resource-constrained settings and age criteria for GC and GT.

Sl. no.	Consensus statement	Agree (%)	Level of consensus
Pre- and post-test GC in resource-constrained settings			
1	Pretest GC and consenting should be offered before ordering a genetic test in BC patients suspected to carry hereditary BC predisposition gene mutations and/or being considered for PARPi treatment.	100	High
2	Pretest GC and consenting can be mainstreamed by trained breast surgeons and/or oncologists or other trained HCPs (including allied HCPs) to reduce the burden on cancer genetics services and to ensure timely access to test results to inform surgical and therapeutic treatment decisions.	87·5	High
3	In general, the ideal time for GT for *BRCA* germline pathogenic variants in *HER2*-negative eBC patients is at the first diagnosis of cancer.	100	High
4	If available, genetic risk assessment tools (e.g. Penn, Myriad, BRCAPRO, BOADICEA, and ARICA) to enrich genetic test positivity rates might be considered in low/under-resourced healthcare systems in order to select patients for GT; however, some patients might be missed from these tools. HCPs may determine thresholds for parameters used in respective risk assessment tools in their respective healthcare systems according to the available resources.	81·3	High
5	Post-test GC should be offered to all patients whose genetic test results are positive. Patients with negative results should be considered for post-test GC as well to avoid wrong interpretation of results, especially where family history remains suggestive of inherited disease.	87·5	High
6	Patients found with likely/pathogenic variants in *BRCA* genes should optimally be referred to a cancer genetics specialist for GC and discussions around screening/prevention and predictive testing of family members.	93·8	High
7	Blood relatives (at least first- and second-degree relatives of the same side of the family) of patients with likely/pathogenic variants in *BRCA* genes should be offered GC and predictive testing.	100	High
Age criteria for GC and GT			
8	In general, GT for *BRCA* germline pathogenic variants should be considered for TNBC patients, irrespective of age at diagnosis or family history.	93·8	High
9	In the absence of access-related challenges, GT for *BRCA* should be offered to HR+*/HER2*-negative eBC patients who may be eligible for adjuvant PARPi treatment, irrespective of age at diagnosis or family history.	87·4	High
10	If age-based selection criteria are to be used in HR+*/HER2*-negative eBC, GT for *BRCA* germline pathogenic variants can be limited to patients aged ≤50 years at cancer diagnosis with an unknown or limited family history of cancer (or ≤45 years of age at cancer diagnosis with no family history of cancer for more restrictive patient selection if there are challenges to access GC/GT).	75	High
11	If selection criteria are to be based on family history of cancer for HR+*/HER2*-negative eBC patients in resource-constrained settings, GT for *BRCA* germline pathogenic variants can be limited to patients with known likely/pathogenic variants in high-penetrance BC susceptibility genes (including *BRCA*1/2) in at least one close blood relative with BC diagnosed at 50 years or less, epithelial ovarian nonmucinous cancer at any age, or high Gleason prostate cancer diagnosed at age less than 60 years.	68·8	Moderate
12	If selection criteria are to be based on the risk of cancer recurrence for HR+*/HER2*-negative eBC patients, GT for *BRCA* germline pathogenic variants should be offered to patients who are assessed to be at high risk of cancer recurrence irrespective of age at cancer diagnosis or family history.	75	High

BC, Breast cancer*;* eBC, Early breast cancer; GC, Genetic counselling; GT, Genetic testing; HCP, Healthcare practitioner; *HER2*, Human epidermal growth factor receptor 2; HR, Hormone receptor; PARPi, Poly (ADP-ribose) polymerase inhibitor; TNBC, Triple-negative breast cancer.

Level of consensus: High ( ≥75%), moderate (55%–74%), low (<55%).

**Table 4:** Level of consensus on tumour BC gene mutation, non-BRCA gene mutation and multigene panel testing, and barriers to GC and GT.

Sl. no.	Consensus statement	Agree (%)	Level of consensus
Tumour BC gene mutation			
13	If a likely/pathogenic germline tumour *BRCA* variants is identified, reflex testing for germline *BRCA*m should be offered to ascertain if the mutation may be germline in nature.	100	High
14	In patients who do not opt for germline testing, tumour *BRCA* (or a gene panel containing *BRCA*) should not be used to make screening/prevention decisions for patients and predictive testing for family members* (N=14).	87·5	High
15	In patients who do not opt for germline testing, tumour *BRCA* (or a gene panel containing *BRCA)* may be considered for PARPi treatment decision for metastatic BC.	87·5	High
16	In patients who do not opt for germline testing, tumour *BRCA* (or a gene panel containing *BRCA*) may be considered for PARPi treatment decision for early-stage BC* (N=14).	35·7	Low
Non-*BRCA* gene mutation and multigene panel testing			
17	Testing for germline *PALB2* mutations might be relevant to inform treatment decisions in high-risk *HER2*-negative eBC patients.	75	High
18	If a multigene panel test is chosen for *HER2*-negative eBC patients for therapeutic indications, the preferred gene panel may include (but not be limited to) *BRCA1* and *BRCA2.*	100	High
19	Individuals carrying pathogenic/likely pathogenic germline variants in *BRCA* (+/− other hereditary genes like *TP53* and *PALB2*) might be considered for risk-reducing surgery.	87·6	High
20	Healthy individuals >25 years old carrying pathogenic/likely pathogenic variants in *BRCA* genes (+/− other hereditary genes like *TP53* and *PALB2*) should undergo BC surveillance with monthly self-examinations, twice-a–year clinical breast examinations, and yearly mammograms (or MRI between age 25 and 35 years if resources permit).	93·8	High
21	Though there is limited clinical evidence, metastatic *HER2*-negative BC patients whose tumours harbour somatic *BRCA* germline pathogenic variants may benefit from PARPi** (N=15).	93·3	High
22	There is robust clinical evidence to demonstrate that PARPi significantly reduces the risk of disease recurrence and provides a clinically meaningful extension of overall survival in *BRCA* germline pathogenic variants carriers with high-risk *HER2*-negative eBC across patient subgroups.	87·5	High
Barriers to GC and GT			
23	Education to the patient, caregivers, and the public to enhance awareness around GT and its implications on the treatment journey (including surveillance for unaffected at-risk healthy individuals) is a shared responsibility between HCPs, the pharmaceutical industry, payors, governments, and patient advocacy groups.	87·5	High

BC, Breast cancer*;* eBC, Early breast cancer; GC, Genetic counselling; GT, Genetic testing; HCP, Healthcare practitioner *HER2*, Human epidermal growth factor receptor 2; MRI, Magnetic resonance imaging; *PALB2*, Partner and localiser of *BRCA2*; PARPi, Poly (ADP-ribose) polymerase inhibitor; *TP53*, Tumour protein 53.

*Statement nos. 14 and 16 included responses from 14 experts.

**Statement no. 21 included responses from 15 experts.

Level of consensus: High ( ≥75%), moderate (55%–74%), low (<55%).

**Table 5:** Level of consensus on the clinical risk assessment for disease recurrence in *HER*2-negative eBC.

Sl. no.	Consensus statement	Agree (%)	Level of consensus
24	Risk assessment for disease recurrence should be performed at diagnosis and after surgery for all *HER*2-negative eBC patients.	93·8	High
25	All eTNBC patients who have undergone upfront surgery and have ≥pT2 or ≥pN1 disease should be considered at high risk of disease recurrence.	93·8	High
26	Any eTNBC patient with *BRCA* germline known likely/pathogenic variants who has undergone upfront surgery may be considered at high risk of disease recurrence, irrespective of tumour size and nodal status.	43·8	Low
27	Any eTNBC patient who has failed to achieve pCR after neoadjuvant treatment and surgery should be considered at high risk of recurrence, regardless of age at diagnosis and/or family history of cancer.	93·8	High
28	Any eTNBC patient with *BRCA* germline known likely/pathogenic variants who has undergone neoadjuvant treatment and surgery and has not achieved pCR status may be considered at high risk of disease recurrence* (N=15).	100	High
29	Rate the following factors on a scale from 0 to 5 based on their relevance to determine the risk of disease recurrence in HR*+/HER*2-negative eBC patients who have undergone upfront surgery (most relevant=5, least relevant=0).	Agree	
a	Histological grade 3	87·5	High
b	Histological type (non-luminal, basal)	75	High
c	≥4 axillary nodal status involved in the pathology	100	High
d	≥3 axillary nodal status involved in the pathology	93·7	High
e	≥2 axillary nodal status involved in the pathology	68·7	Moderate
f	Primary tumour size >5 cm	93·7	High
g	Primary tumour size >2 cm	49·9	Low
h	Ki-67 >30%	81·2	High
i	Ki-67 >20%	49·9	Low
j	Absence or <20% PgRs	49·9	Low
k	Low level of ERs (<1%)	74·9	High
l	Residual cancer burden score 3 (after neoadjuvant therapy)	87·4	High
m	High Oncotype DX^®^/Mammaprint^®^/Prosigna/Endopredict^®^ scores	100	High
n	Intermediate Oncotype DX^®^/Mammaprint^®^/Prosigna/Endopredict^®^ scores	43·7	Low
30	It is unclear whether HR+/*HER*2*-*negative eBC patients who have undergone neoadjuvant therapy but who did not achieve pCR may/may not be categorised as patients at high risk of disease recurrence* (N=15).	73·3	Moderate
31	In HR*+/HER*2-negative eBC, patient selection should be guided by the trial eligibility criteria but the physician’s discretion to clinical judgement may be used to identify selective high-risk cases to decide on the use of adjuvant PARPi* (N=15).	86·7	High
32	In HR*+/HER2*-negative eBC, disease recurrence risk assessment tools (e.g. CPS + EG score, Oncotype DX^®^, and MammaPrint^®^) could be used to support treating physicians’ clinical judgement and adjuvant treatment decisions.	93·8	High
33	Young age at cancer diagnosis is considered an independent factor in determining the risk of disease recurrence in both eTNBC and HR*+/HER*2-negative eBC.	81·3	High

CPS + EG, Clinical and pathologic stage and oestrogen receptor status and histologic grade; eBC, Early breast cancer; ER, Oestrogen receptor; eTNBC, Early triple-negative breast cancer; *HER*2, Human epidermal growth factor receptor-2; HR, Hormone receptor; PARPi, Poly ADP-ribose polymerase inhibitor; pCR, Pathological complete response; PgRs, Progesterone receptors.

*Statement nos. 28, 30, and 31 included responses from 15 experts.

Level of consensus: High ( ≥75%), moderate (55%–74%), low (<55%).

### 3.1 GC and GT in *HER2*-negative eBC patients for surgical and therapeutic decisions

#### 3.1.1 Pre- and post-test GC in resource-constrained settings

The statements reached high consensus on pretest GC and consent before GT; mainstreaming GC and consent by trained HCPs; emphasising the ideal timing of GT; using genetic risk assessment tools in low-resource settings; offering post-test GC, referring patients with likely/pathogenic *BRCA* gene variants to specialists; and testing their blood relatives (Statements 1 to 7, **Table 3**).

#### 3.1.2 Age criteria for GC and GT

The statements reached high consensus for considering GT for patients with TNBC and hormone receptor-positive (HR+)/*HER2*-negative eBC eligible for adjuvant poly (ADP-ribose) polymerase inhibitor (PARPi) treatment irrespective of age; considering age-based selection criteria for offering GT in case of no/limited family history of BC; and offering GT based on cancer recurrence risk. A moderate consensus was reached for considering family history-based selection criteria for offering GT in resource-constrained settings (Statements 8 to 12, **Table 3**).

#### 3.1.3 Tumour BC gene mutation

The statements reached high consensus for offering reflex testing for patients with likely/pathogenic germline *BRCA* variant; not considering tumour *BRCA* for screening, prevention decisions of patients, or predictive testing of family members; and considering tumour *BRCA* for PARPi treatment decision in patients with metastatic BC who decline germline testing. A low consensus was reached for considering tumour *BRCA* for PARPi treatment decision in patients with early-stage BC (Statements 13 to 16, **Table 4**).

#### 3.1.4 Non-*BRCA* gene mutation and multigene panel testing

The statements reached high consensus for testing for germline *PALB2* mutations and including *BRCA1* and *BRCA2* genes in the gene panel for therapeutic indications in high risk *HER2*-negative eBC patients; considering patients with likely/pathogenic variants in *BRCA* for risk reduction surgery; undergoing regular BC surveillance, including self-examinations and clinical breast exams in healthy carriers; providing benefits from PARPi for metastatic patients with somatic *BRCA* pathogenic variants (Statements 17 to 22, **Table 4**).

#### 3.1.5 Barriers to GC and GT

The statement reached high consensus for increasing awareness on GT, having shared responsibilities, and surveillance of at-risk individuals (Statement 23, **Table 4**).

### 3.2 Clinical risk assessment for disease recurrence in *HER2*-negative eBC

The statements reached high consensus for initiating risk assessment at diagnosis and continuing it after surgery; considering patients at high risk based on tumour size and lymph node involvement; considering early triple-negative breast cancer (eTNBC) with known *BRCA* germline pathogenic variants at high risk irrespective of tumour size and lymph node involvement; considering patients with eTNBC or eTNBC with *BRCA* mutations at high risk who do not achieve pathological complete response (pCR) after neoadjuvant treatment and surgery. High consensus was also reached for adhering to trial eligibility criteria for patient selection for adjuvant PARPi; utilising disease recurrence risk assessment tools for making informed decisions about adjuvant treatment; and considering young age as independent risk factor for recurrence in eTNBC and HR+/*HER2*-negative eBC. A moderate consensus was reached for the statement that was unclear in terms of classifying patients with HR+/*HER2*-negative eBC at high risk of recurrence who do not achieve pCR after neoadjuvant therapy (Statements 24 to 33, **Table 5**).

### 3.3 HCP survey

A total of 134 HCPs took part in the survey. Though the response rate was low (35·3% [134/380]), which could be attributed to the busy schedules of the responders, there was a fair representation from multidisciplinary members experienced in managing BC across various Asian countries. Additionally, it should be noted that the survey was conducted only to highlight the unmet needs and gaps in practice and was not used to refine or modify the final recommendations. Therefore, the low response rate did not impact generalisability. The responses obtained in the survey are presented in **Supplementary Table S4**. The participants were from Australia (9·0%), India (5·2%), Indonesia (2·2%), Malaysia (21·6%), the Philippines (12·7%), South Korea (6·7%), Singapore (8·2%), Taiwan (11·9%), Thailand (9·0%), and Vietnam (13·4%). The surveyed HCPs were breast surgeons (24·6%), oncologists (65·7%), geneticists (8·2%), and molecular biologists/pathologists (1·5%); 56·7% of the HCPs were from the public sector and 42·5% were from the private sector, and 0·7% of the HCPs practised in both sectors (**Supplementary Figures S1**, **S2**).

A significant gap was observed between real-world practices and the recommendations of the SCMs in offering pretest GC to patients with suspected hereditary BC (HCPs [54·5%] vs SCMs [100%]) and blood relatives of patients with *BRCA* germline pathogenic variant BC (HCPs [70·9%] vs SCMs [100%]). There were differences in opinion between the SCMs and the HCPs regarding practical scenarios, such as GT for patients with *BRCA* germline pathogenic variants at the first diagnosis of BC (HCPs [47·8%] vs SCMs [100%]) and GT for patients with eTNBC (HCPs [37·3%] vs SCMs [93·8%]) and with HR+*/HER2*-negative eBC (HCPs [39·6%] vs SCMs [87·4%]) irrespective of age. Additionally, there were differences in agreement regarding the inclusion of clinical and pathologic stages and oestrogen receptor status and histologic grade (CPS + EG) scores in the risk assessment tools (HCPs [67·9%] vs SCMs [93·8%]), discussions about risk-reducing mastectomy (RRM) with patients (HCPs [41·8%] vs SCMs [87·5%]), and preference for streamlining GC through non-geneticist HCPs (HCPs [48·5%] vs SCMs [87·5%]). Multiple reasons for the suboptimal implementation of GT by real-world practitioners in the Asia-Pacific region included the lack of available funding for GT and, subsequently, the high cost to the patient for GT (82·1%), the high cost of treatment following the test (66·4%) in the setting of identification of a pathogenic variant or likely pathogenic variant lack of genetic counsellors for GC in the region (58·2%), low patient awareness about GT (49·3%), and lack of multidisciplinary discussions (27·6%). The results of the real-world questionnaire are discussed and compared wherever applicable in the discussion section. These results are further discussed under the same domains with supporting literature in the subsequent section.

## 4 Discussion

### 4.1 GC and GT in *HER2*-negative eBC patients for surgical and therapeutic decisions

Approximately, 5%–5·7% of all BCs can be linked to pathogenic variants in 12 actionable genes associated with BC risk. Of these, <50% (absolute rate: 2·1%–2·6%) were associated with *BRCA1*/*2* genes, highlighting that even when multiple genes are tested, *BRCA1/2* remain the most significant contributors to BC risk (10, 12). The management of hereditary BC does not differ in Asian and European women as there are no differences in the prevalence of well-defined moderate-to-high penetrance genes associated with BC risk (12). It is crucial to improve the comprehension of the variant spectrum in these populations to improve the rates of early diagnosis and management of hereditary BC in Asia-Pacific region (23).

#### 4.1.1 Pre- and post-test GC in resource-constrained settings

Only 54·5% of the surveyed HCPs considered that it is important to do pretest GC and consenting of BC patients suspected to carry hereditary BC, 10·4% mentioned that they offered counselling after the test result was available, and another 10·4% did not provide counselling at all (**Supplementary Table S4**, Q1). These results could be due to a lack of resources in the real world as well as a lack of awareness regarding GC/GT among the HCPs. Additionally, social and cultural factors may also play a role. In many parts of the world, testing, particularly germline testing, would not be acceptable without prior comprehensive consent obtained through GC. On the other hand, there was a unanimous consensus among the SCMs that recommending GC is important for patients with hereditary BC (Statement No. 1). The recommendations of the SCMs aligned with those of the American Society of Clinical Oncology–Society of Surgical Oncology (ASCO–SSO) guidelines, emphasising the importance of ensuring patients receive sufficient information before GT to obtain informed consent (24). All SCMs agreed that the best time to administer GT is at the time of diagnosis for *HER2*-negative eBC patients (Statement No. 3), but less than half (47·8%) of the HCPs concurred with this (**Supplementary Table S4**, Q3). In the real-world survey of the present study, 83·6% of the HCPs agreed that GC could be provided by non-geneticists who have undergone proper training (**Supplementary Table S4**, Q2), and 48·5% opined that GC could be streamlined through non-geneticist HCPs in their countries (**Supplementary Table S4**, Q13). Similarly, the majority of the SCMs (87·5%) agreed that other trained HCPs could carry out pretest GC (considering the increasing incidence and mortality due to BC in the Asia-Pacific region) (Statement No. 2) and recommended that written informed consent from the patients is required after discussing the risks, benefits, and limitations of the testing options with the patients. International guidelines support this statement (19, 25, 26).

The panel agreed that in a resource-constrained setting, evaluating the likelihood of having a germline pathological variant is necessary to ascertain the risks and advantages of GT using risk-predictive tools. However, the threshold risk rate could be set at the HCPs’ discretion to decide whether patients qualify for GT (Statement No. 4) (23, 27). The SCMs reached a consensus for post-test GC for all patients (Statement No. 5), which is also supported by National Comprehensive Cancer Network^®^ (NCCN) guidelines. NCCN guidelines recommend post-test GC regardless of the actual result and can be done in person or remotely. Additionally, the results should be interpreted in conjunction with personal and family history of cancer (25). The agreement of the SCMs was also supported by ASCO–SSO, which recommends that patients be provided with post-test GC and appropriate referrals to cancer genetics in case of a positive test. Patients with no pathogenic variants on GT can still benefit from GC, particularly if they have a family history of cancer (24).

#### 4.1.2 Age criteria for GC and GT

The epidemiology of BC is different for women in Asian countries compared to women in Western countries. Additionally, *BRCA*-associated BCs are seen at younger ages among women in Asian countries compared to women in Western countries (11). The SCMs recommended GT for all HR+*/HER2*-negative eBC patients who may be eligible for adjuvant PARPi treatment, irrespective of age at diagnosis or family history (87·4% agreement; Statement No. 9). Eligibility for adjuvant PARPi treatment typically includes patients who have a germline *BRCA1* or *BRCA2* pathogenic or likely pathogenic variants and have high-risk, *HER2*-negative primary BC after definitive local treatment and neoadjuvant or adjuvant chemotherapy. Further, eligibility includes patients with TNBC treated with adjuvant chemotherapy who have an axillary node-positive disease or an invasive primary tumour of at least 2 cm. For those treated with neoadjuvant chemotherapy, residual invasive BC in the breast or resected lymph nodes is required (non-pCR). Patients with HR+/*HER2*-negative BC treated with adjuvant chemotherapy need at least four pathologically confirmed positive lymph nodes. Those treated with neoadjuvant chemotherapy require a non-pCR and a CPS+EG score of ≥3 (28, 29). However, in the real-world survey, only 39·6% of the HCPs agreed with this statement (**Supplementary Table S4**, Q4).

In the real-world survey, 68·7% of the HCPs selected HR+ patients aged ≤50 years with family history and/or at a high risk of recurrence for GT (**Supplementary Table S4**, Q4). There was a similar high consensus among the SCMs (75%) on the age criteria of ≤50 years at cancer diagnosis or ≤45 years for more restrictive patient selection (Statement No. 10). The primary purpose of this statement was to have a set of more stringent selection criteria, considering resource limitations in some countries. The panel voiced that other groups of patients who do not fulfil these restricted clinical criteria could be at high risk of recurrence. Hence, the criteria should not be strictly enforced if resources permit. One of the panellists stated that subjects in the OlympiA trial had a median age of about 45 years and suggested that testing should be focussed on younger women in a resource-constrained setting.

About 68·8% of the SCMs opined that, if selection criteria are to be based on family history of cancer for HR+/*HER2*-negative eBC patients in resource-constrained settings, GT for *BRCA* germline pathogenic variants can be limited to patients with known likely/pathogenic variants in high-penetrance BC susceptibility genes (including *BRCA1/2*) in at least one close blood relative with BC diagnosed at age ≤50, epithelial ovarian non-mucinous cancer at any age, or high Gleason prostate cancer diagnosed at age <60 years (Statement No. 11). ASCO–SSO guidelines recommend considering *BRCA1*/2 germline pathogenic variants testing for all individuals diagnosed with BC up to the age of 65, with selective testing for those above 65 based on personal and family history as well as ancestry (24).

#### 4.1.3 Tumour BC gene mutation

The panellists acknowledged that tumour next-generation sequencing (NGS) is increasingly being used in practice. However, oncologists should be aware that variants identified by tumour sequencing may represent incidental germline findings. In particular, the detection of tumour pathogenic variants in *BRCA1/2* should trigger the referral of the patient for GC and germline testing if resources are available (Statement No. 13) (26). The panel acknowledged that there are multiple reasons in the real world for patients not opting for germline testing. Nonetheless, one of the panellists expressed that tumour testing is not a surrogate marker for delineating germline risk. Tumour *BRCA* can help in treatment planning in selected situations; however, the SCMs would not endorse using tumour *BRCA* test results to make screening decisions for patients and predictive testing decisions for family members. The majority of the SCMs agreed to this (Statement No. 14). Furthermore, the panellists discussed that germline GT has become less costly and offered by more labs and should become more accessible to patients (19).

Most tumour *BRCA* germline pathogenic variant findings reflect a germline predisposition (30). However, it was expressed by the SCMs that it is not standard practice to use tumour *BRCA* results as a part of treatment planning. Few landmark trials and the United States Food and Drug Administration (US FDA) used *BRCA* germline pathogenic variants as the biomarker to select patients for PARPi treatment. The SCMs opined that it could be acceptable to use PARPi for metastatic *HER2*-negative BC based on tumour *BRCA* pathogenic variants, whereas there was low agreement on its use in *HER2*-negative eBC cases (Statement Nos. 15 and 16). This remains a controversial topic. Of note, in the, 2023 St. Gallen consensus panel meeting, 48% of the panellists agreed that they would offer olaparib to patients with BC having a somatic *BRCA1* pathogenic variant but no hereditary germline mutation, whereas 47% of the panellists mentioned that they would not (31).

#### 4.1.4 Non-*BRCA* gene mutation and multigene panel testing

Among non-*BRCA1/2* high-penetrance BC predisposition genes, the partner and localiser of the *BRCA2* (*PALB2)* gene is one of the most common, after *BRCA1* and *BRCA2* (32). There is preliminary evidence of the efficacy of PARPi in patients with metastatic BC carrying germline *PALB2* mutations (33). The SCMs strongly agreed that testing for germline *PALB2* mutations could be informative in managing high-risk *HER2*-negative eBC (Statement No. 17). In contrast, the expert panel in the, 2023 St. Gallen consensus report reached only a low consensus (38%) on offering adjuvant PARPi to germline *PALB2* mutation carriers (31).

When asked to choose a multigene panel test for therapeutic indications in a resource-constrained healthcare system, various factors, such as accessibility, affordability, and manpower, were considered. The SCMs expressed that most cases of germline mutant BCs in the Asia-Pacific region are due to mutations in *BRCA1/2*; however, there is a lack of evidence for the involvement of other genes owing to the low rates of germline testing. A few panellists opposed the concept of including a multigene panel, as obtaining test results from the laboratory would take time and hamper the mainstreaming delivery model for non-genetic specialists in many resource-deficient countries. In Vietnam and Malaysia, germline testing is restricted to *BRCA1/2* due to the unaffordability of NGS. Furthermore, they are more focussed on GT for treatment purposes. The panel suggested that if the cost of the NGS panel is affordable, the oncologists should be ready to include it in their practice, with appropriate pre-test genetic counselling. However, if affordability is an issue, the preferred gene panel may include *BRCA1* and *BRCA2* only for therapeutic purposes (Statement No. 18). The panel agreed that while there is limited evidence for the efficacy of PARPi in *HER2*-negative metastatic BC patients with tumours harbouring somatic *BRCA* pathogenic variants, this is in part due to the paucity of studies involving BC patients with deleterious somatic mutations of *BRCA1/2* (Statement No. 21).

#### 4.1.5 Barriers to GC and GT

The lack of patient awareness as a barrier for GT was cited by around 50% of the HCPs in the real-world survey and was also agreed upon by 87·5% of the SCMs. Hence, there is a high consensus among the SCMs that steps to increase awareness about GC and GT are a shared responsibility of all the members involved in the holistic management of BC (Statement No. 23). Further, the HCPs also highlighted barriers such as lack of genetic counsellors leading to long waiting time, lack of MDT discussions, and cost of GT and treatment in implementing GT in Asia-Pacific region. These barriers are particularly encountered in low or under-resource countries. A systematic review aimed at uncovering the issues related to the implementation of GC and GT in resource-limited countries for various disorders including cancer also highlighted similar barriers as that reported by the HCPs of the present study (18). Additional barriers reported were GC to be directive, the psychosocial consequences of genetic services necessitating improved support, inadequate medical genetics training, and difficulties in accessing genetic services (18). Sociocultural barriers are also evident in the Asia-Pacific region that limit the uptake of genetic services such as GC and GT. These barriers include the impact of social determinants on the awareness and acceptance of genetic services, concerns about potential disruptions to family values, religious principles limiting their acceptability and use, and cultural beliefs and practices that influence the understanding and uptake of genetic information (18). A study among Malay women reported that they considered breast cancer screening as taboo due to the need to expose breasts which is culturally prohibited. Women considered GT as ongoing research, presented a lack of trust in the procedure, and preferred traditional medicine practitioners. Further, women expressed that GT involved multiple family members and the cost of GT along with the treatment was viewed as an emotional and financial burden to the family, limiting them from seeking this care (34). Nevertheless, a systematic review reported that, both *BRCA* and multigene testing (including *BRCA1/BRCA2/PALB2*) are cost-effective compared to no testing or limited testing in upper-middle-income countries like China, Brazil, and Malaysia with incremental cost-effectiveness ratios (ICERs) ranging from United States Dollar (USD) 2214/quality-adjusted life year (QALY) to USD 36,342/QALY. Multigene testing in BC patients with cascade testing showed an ICER of USD, 7729/QALY. However, in lower-middle-income countries like India, *BRCA* testing for population-based screening in women aged ≥30 years would not be cost-effective, with ICERs of USD ranging between 36,342/QALY and USD 25,980/QALY (35). The barriers to GC and GT can be overcome by adopting some innovative services such as telegenetics. Various health models can be utilised such as the use of the telephone (36), and real-time two-way videoconferencing (RTVC) for counselling patients on breast cancer (Sim J et al., 2021). The study by Chin XW et al. (2020) highlighted that telephone-based GC increases accessibility, and given its feasibility, this can serve to be important in countries with vast geographical areas, making it a feasible alternative service delivery model for rural and remote regions (36). Likewise, a study by Sim J et al. (2021) demonstrated patient’s willingness to receive counselling via telephone or videoconference, noting that these methods would effectively meet their needs for understanding the information, asking questions, and arranging follow-up visits. However, concerns were raised about the adequacy of emotional support provided through these approaches (37). Further, gynaecology and primary care clinics can be used to improve the delivery of GC since these are the centres often visited by women even at young ages before the onset of breast cancer (38).

### 4.2 Clinical risk assessment for disease recurrence in *HER2*-negative eBC

Despite well-recognised clinical and pathological risk factors and prognostic staging, there is no standard definition of “high risk” for patients with *HER*2-negative eBC. A better understanding of the recurrence risks can help HCPs identify and treat patients who may require additional therapy while avoiding overtreatment in low-risk patients (39).

The majority of the SCMs agreed that all patients with eTNBC who have undergone upfront surgery and have ≥pT2 or ≥pN1 disease should be considered as being at high risk of disease recurrence. The statement is supported by the OlympiA trial inclusion criteria (Statement No. 25). Half of the SCMs did not agree that any patient with eTNBC with germline *BRCA* known likely/pathogenic variants who has undergone upfront surgery may be considered at high risk of disease recurrence, irrespective of tumour size and nodal status. It was highlighted that not all patients with eTNBC with *BRCA* germline pathogenic variants are at high risk of disease recurrence (Statement No. 26). There is high consensus among the SCMs that histological grade, histological type, nodal involvement, tumour size, Ki-67 labelling index, and genomic signatures are important factors that influence the risk of relapse of surgically resected HR*+/HER*2-negative eBC (Statement No. 29). The response of the SCMs is supported by a consensus review reported by Garutti et al. (2022) (40).

There is a high consensus (87·5%) among the SCMs that the selection of high-risk HR+*/HER2*-negative eBC for adjuvant olaparib should be guided by the trial eligibility criteria after neo/adjuvant chemotherapy, although the physician’s discretion in clinical judgement may be used to identify selective high-risk cases. One of the panellists suggested that some high-risk patients do not fall within the inclusion criteria of the OlympiA trial but are at high risk for recurrence and may potentially benefit from adjuvant olaparib. All the SCMs agreed that the OlympiA trial inclusion criteria should be used as a guiding reference (Statement No. 31), although it was noted that the US FDA-approved adjuvant olaparib for high-risk *HER*2*-*negative eBC without an explicit definition of what constitutes “high risk.” In Asia-Pacific region, olaparib is approved in Japan for the adjuvant treatment of patients with *BRCA*-mutated, *HER2*-negative eBC at high risk of recurrence (41).

One of the limitations of this study is the relatively low response rate from HCPs to the real-world survey questionnaire which could limit the generalisability of the findings and recommendations. Nevertheless, it is important to highlight that the HCPs who responded, represented a diverse group from ten countries across the Asia-Pacific region, including both developed and developing nations such as Australia, India, Indonesia, Malaysia, the Philippines, South Korea, Taiwan, Singapore, Thailand, and Vietnam, ensuring that the study findings are applicable and relevant across various healthcare systems, cultures, and patient populations within the region. Another limitation is the discrepancy among experts due to limited resources and differing ethnic backgrounds, which may have influenced the findings. In addition, while consensus was achieved for 88% of the statements, the underlying reasons for the lower agreement on specific contentious topics (e.g., tumour *BRCA* testing) require further exploration. Additionally, the recommendations may face significant practical implementation challenges, particularly in low-resource settings where integrating GC through non-geneticists may prove difficult.

## 5 Conclusion

**Figure 3:**
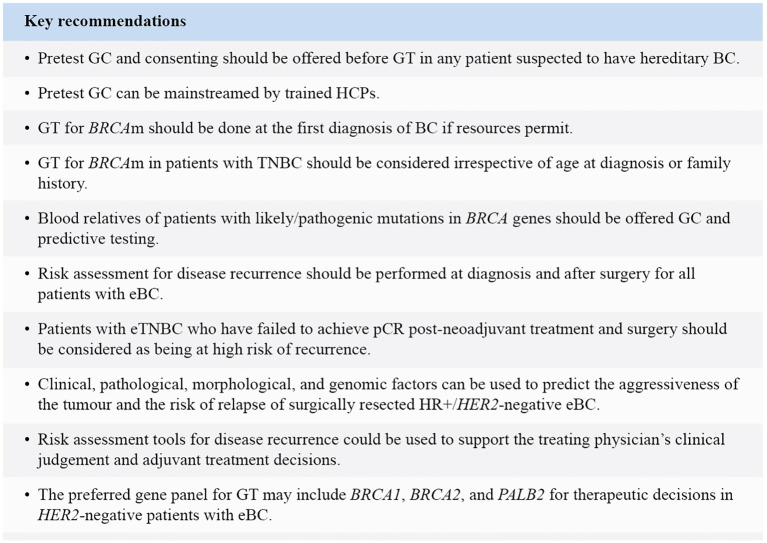
Key recommendations. BC, Breast cancer; eBC, Early breast cancer; eTNBC, Early triple-negative breast cancer.*GC, Genetic counselling; GT, Genetic testing; *HER*2, Human epidermal growth factor receptor 2; *HR*, Hormone receptor; m, Mutation; *PALB*2, Partner and localiser of *BRCA*2; pCR, Pathological complete response; TNBC, Triple-negative breast cancer.

The outcome of three rounds of the modified Delphi technique resulted in high consensus for most of the statements. This consensus paper considers the ethnic and geographical differences and resource availabilities associated with managing BC in Asian populations. It highlights the unmet needs for treatment/GT/GC of *BRCA-*associated BC in Asia-Pacific, thus highlighting the need for making more resources available for GC/GT. It emphasises the need for early *BRCA* germline pathogenic variants testing with priority criteria in resource-constrained healthcare systems and inclusive clinical definitions of “high risk” guided by clinical studies and physician judgement. HCPs with appropriate training should consider mainstreaming pretest GC/GT at the first diagnosis of breast cancer. Risk assessment for disease recurrence should be performed at diagnosis and after surgery for patients with eBC. These recommendations are believed to aid in homogenising GC and stratifying high-risk patients, thereby improving surgical and therapeutic decisions. This paper opens new vistas for future research. Future studies or pilot programs are warranted to validate the practical implementation of these recommendations in diverse healthcare settings across the Asia-Pacific region. These initiatives will help identify and address potential barriers, ensuring effective implementation and integration into clinical practice. The studies should also take into consideration the challenges of ethical aspects for implementation of GC and GT in Asia-Pacific regions. The key recommendations of the SCMs are summarised in **Figure 3**.
