# Supplementary figures and images for: Part I: consensus statements and expert recommendations for HER2-negative early breast cancer in the Asia-Pacific region: diagnosis and risk assessment
Source: Front Oncol. 2025 Jun 23;15:1507836. doi: 10.3389/fonc.2025.1507836 (PMC12230085; doi:10.3389/fonc.2025.1507836)

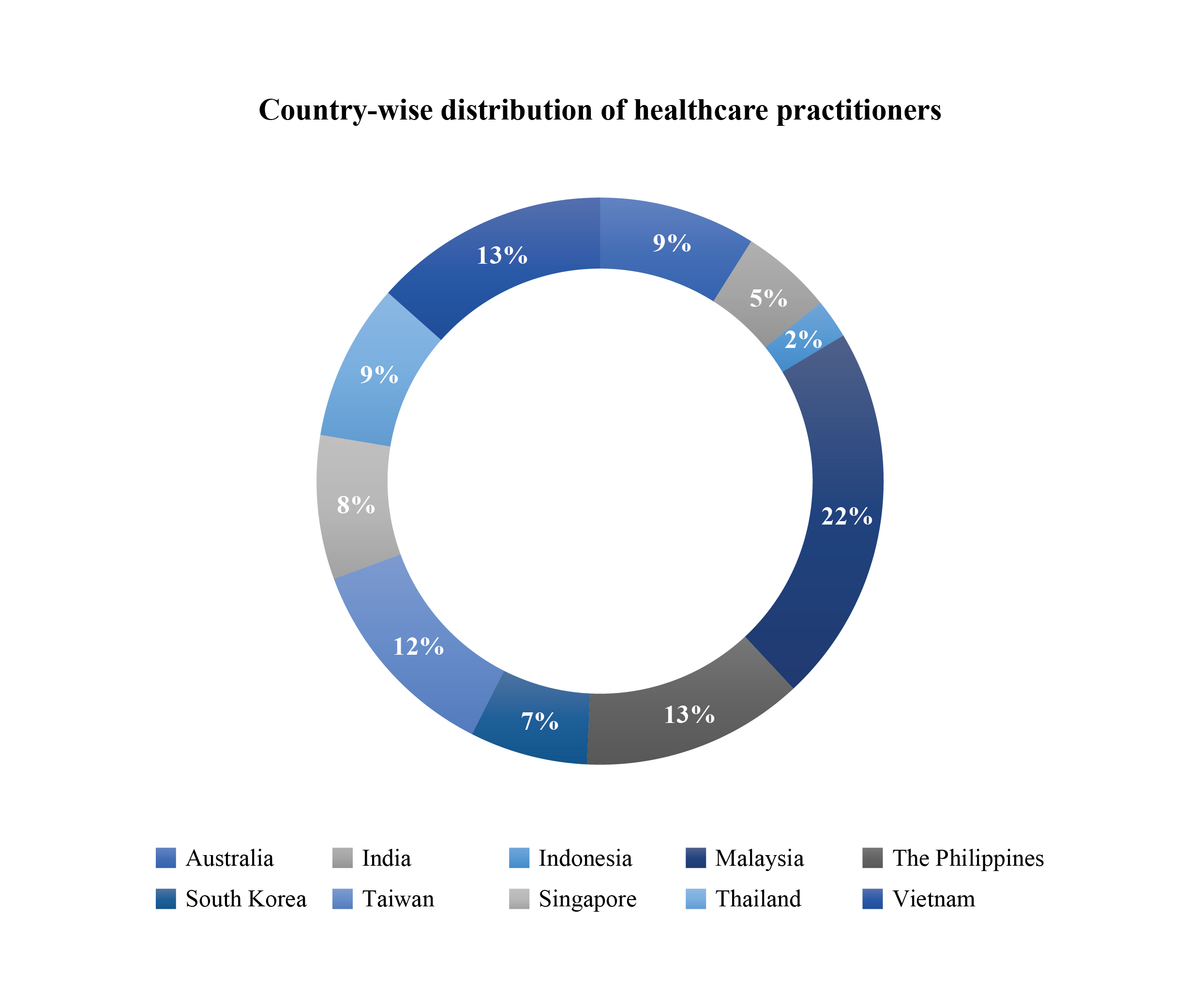

Supplement: Supplementary file 1 [file Image1.jpeg]

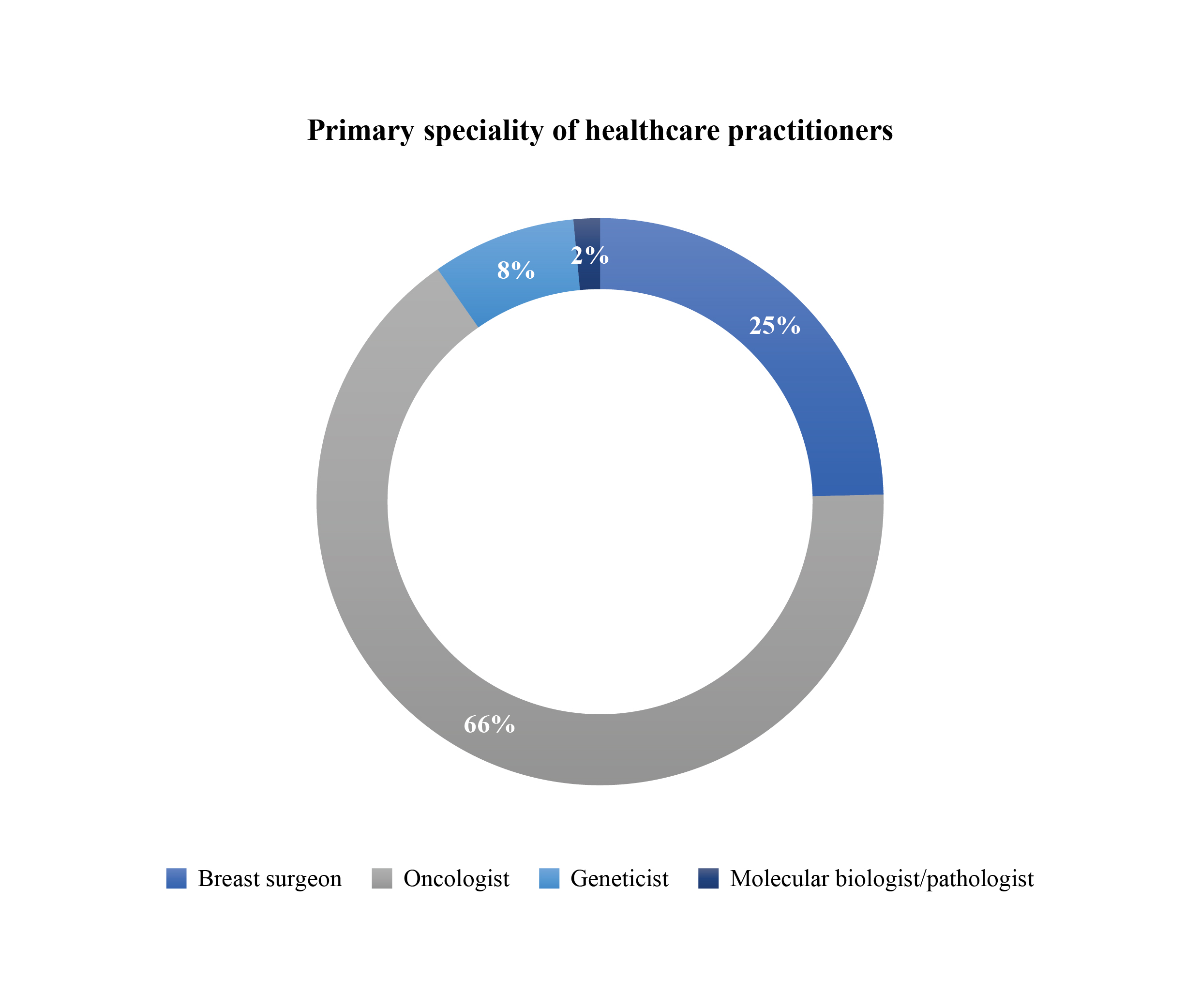

Supplement: Supplementary file 2 [file Image2.jpeg]
