# Supplementary material for: Part I: consensus statements and expert recommendations for HER2-negative early breast cancer in the Asia-Pacific region: diagnosis and risk assessment
Source: Front Oncol. 2025 Jun 23;15:1507836. doi: 10.3389/fonc.2025.1507836 (PMC12230085; doi:10.3389/fonc.2025.1507836)
Supplement: Supplementary file 3 [file Table1.docx]

**Supplementary Material**

**Supplementary Table S1.** Expert panel for the consensus

| **Name of the member** | **Specialty** | **Place** |
| --- | --- | --- |
| Prof. Lee Soo Chin (Co-chair) | Medical Oncologist/Geneticist | Singapore |
| Prof. Christian Singer (Co-chair) | Medical Oncologist | Austria |
| Prof. Judith Balmana | Medical Oncologist | Spain |
| A/Prof. Rebecca Dent | Medical Oncologist | Singapore |
| Dr. Veronique Tan | Breast Surgeon | Singapore |
| A/Prof. Yoland Antill | Medical Oncologist/Geneticist | Australia |
| Dr. Nadia Ayu Mulansari | Medical Oncologist | Indonesia |
| Dr. Mastura Md Yusof | Medical Oncologist | Malaysia |
| Dr. Frances Victoria Que | Medical Oncologist | Philippines |
| Prof. Yeon Hee Park | Medical Oncologist | S. Korea |
| Prof. Yen Shen Lu | Medical Oncologist | Taiwan |
| Assoc. Prof. Napa Parinyanitikul | Medical Oncologist | Thailand |
| Assoc. Prof. Pham Cam Phuong | Medical Oncologist | Vietnam |
| Prof. Nur Aishah Taib | Breast Surgeon/Geneticist | Malaysia |
| Prof. Sun Young Kong | Geneticist | S. Korea |
| Prof. Hee Jeong Kim | Breast Surgeon | S. Korea |

**Supplementary Table S2.** Search strategy used for literature search

Search limit: Up to 31 October 2022

| **Search no.** | **Search string** |
| --- | --- |
| **PubMed** | |
| **#1** | ("Breast Neoplasms"[MeSH Terms] OR breast cancer[MeSH Terms] OR "Breast Cancer"[Title/Abstract] OR "Breast Carcinoma"[Title/Abstract] OR “early breast cancer” OR "Malignant Breast Tumor"[Title/Abstract] OR "Invasive Breast Cancer"[Title/Abstract] OR "Non-Invasive Breast Cancer"[Title/Abstract] OR "Ductal Carcinoma In Situ"[Title/Abstract] OR "Lobular Carcinoma In Situ"[Title/Abstract] OR "Triple-Negative Breast Cancer"[Title/Abstract] OR “human epidermal growth factor receptor 2” [Title/Abstract] OR "HER2-negative Breast Cancer"[Title/Abstract] OR “HR-positive”[All Fields] OR "Metastatic Breast Cancer"[Title/Abstract] OR "BRCA1 Protein"[MeSH Terms] OR "Genes, BRCA1"[MeSH Terms] OR "Genes, BRCA2"[MeSH Terms] OR "breast cancer susceptibility"[Title/Abstract] OR "hereditary breast cancer"[Title/Abstract] |
| **#2** | "DNA testing"[Title/Abstract] OR "genomic testing"[Title/Abstract] OR "mutation screening"[Title/Abstract]) OR ("Diagnosis"[MeSH Terms] OR “genomic stability"[Title/Abstract] OR "cancer risk assessment”[Title/Abstract] OR "diagnosis"[Title/Abstract] OR "diagnostic assessment"[Title/Abstract] OR "early detection"[Title/Abstract] OR "early diagnosis"[Title/Abstract] OR "Genetic Testing"[MeSH Terms] OR "Genetic Counseling"[MeSH Terms] |
| **#3** | "Clinical Management"[Title/Abstract] OR "Patient Care Management"[MeSH Terms] OR "treatment sequencing"[Title/Abstract] OR "patient care management"[Title/Abstract] OR "treatment planning"[Title/Abstract] OR "clinical guidelines"[Title/Abstract] OR "management guidelines"[Title/Abstract] OR "treatment guidelines"[Title/Abstract] OR "therapeutic strategies"[Title/Abstract] OR "clinical decision making"[Title/Abstract] OR "multidisciplinary care"[Title/Abstract] OR "personalized medicine"[Title/Abstract] OR "targeted therapy"[Title/Abstract] OR "chemotherapy"[Title/Abstract] OR "radiation therapy"[Title/Abstract] OR "surgical options"[Title/Abstract] OR "hormonal therapy"[Title/Abstract] OR "immunotherapy"[Title/Abstract] OR "palliative care"[Title/Abstract] |
| **#4** | "Risk Assessment"[MeSH Terms] OR "risk assessment"[Title/Abstract] OR "risk analysis"[Title/Abstract] OR "risk evaluation"[Title/Abstract] OR "risk factors"[Title/Abstract] OR "cancer risk factors"[Title/Abstract] OR "breast cancer risk"[Title/Abstract] OR "predictive modeling"[Title/Abstract] OR "prognostic factors"[Title/Abstract] OR "cancer prediction models"[Title/Abstract] OR "cancer prognosis"[Title/Abstract] OR "survival analysis"[MeSH Terms] OR "survival rate"[Title/Abstract] OR "genetic predisposition to disease"[MeSH Terms] OR "genetic risk"[Title/Abstract] OR "family history"[Title/Abstract] OR "hereditary risk"[Title/Abstract] OR "lifestyle risk factors"[Title/Abstract] OR "environmental risk factors"[Title/Abstract] |
| **#5** | "Recurrence"[MeSH Terms] OR "cancer recurrence"[Title/Abstract] OR "breast cancer recurrence"[Title/Abstract] OR "local recurrence"[Title/Abstract] OR "regional recurrence"[Title/Abstract] OR "distant recurrence"[Title/Abstract] OR "relapse"[Title/Abstract] OR "cancer relapse"[Title/Abstract] OR "breast cancer relapse"[Title/Abstract] OR "recurrent breast cancer"[Title/Abstract] OR "tumor recurrence, local"[MeSH Terms] OR "second primary neoplasms"[MeSH Terms] OR "secondary cancer"[Title/Abstract] OR "metachronous neoplasms"[Title/Abstract] OR "second primary cancer"[Title/Abstract] |
| **#6** | “Asia-Pacific”[All Fields] OR “Afghanistan”[MeSH Terms] OR “Australia”[MeSH Terms] OR “Bangladesh”[MeSH Terms] OR “Bhutan”[MeSH Terms] OR “Burma”[All Fileds] OR “Brunei”[All Fields] OR “Cambodia”[MeSH Terms] OR “China”[MeSH Terms] OR “Cook Islands”[MeSH Terms] OR “Federated States of Micronesia”[MeSH Terms] OR “Fiji”[MeSH Terms] OR “India”[MeSH Terms] OR “Indonesia”[MeSH Terms] OR “Japan”[ MeSH Terms] OR “Kiribati”[MeSH Terms] OR “Laos”[MeSH Terms] OR “Malaysia”[MeSH Terms] OR “Maldives”[MeSH Terms] OR “Marshall Islands”[MeSH Terms] OR “Mongolia”[MeSH Terms] OR “Nepal”[MeSH Terms] OR “New Caledonia”[MeSH Terms] OR “New Zealand”[MeSH Terms] OR “Niue”[MeSH Terms] OR “North Korea”[All Fields] OR “Pakistan”[MeSH Terms] OR “Palau”[MeSH Terms] OR “Papua New Guinea”[MeSH Terms] OR “Philippines”[MeSH Terms] OR “Singapore”[MeSH Terms] OR “Solomon Islands”[MeSH Terms] OR “South Korea”[MeSH Terms] OR “Sri Lanka”[MeSH Terms] OR “Taiwan”[MeSH Terms] OR “Thailand”[MeSH Terms] OR “Timor-Leste” [MeSH Terms] OR “Tonga”[MeSH Terms] OR “Tuvalu”[MeSH Terms] OR “Vanuatu”[MeSH Terms] OR “Vietnam”[MeSH Terms] |
| **#7**  **(Diagnosis and Management)** | #1 AND #2 AND #3 AND #6 |
| **#8**  **(Risk assessment and recurrence)** | #1 AND #4 AND #5 AND #6 |
| **Cochrane** | |
| **#1** | ([mh "Breast Neoplasms"] OR [mh "breast cancer"] OR "Breast Cancer":ti,ab OR "Breast Carcinoma":ti,ab OR "early breast cancer" OR "Malignant Breast Tumor":ti,ab OR "Invasive Breast Cancer":ti,ab OR "Non-Invasive Breast Cancer":ti,ab OR "Ductal Carcinoma In Situ":ti,ab OR "Lobular Carcinoma In Situ":ti,ab OR "Triple-Negative Breast Cancer":ti,ab OR "human epidermal growth factor receptor 2":ti,ab OR "HER2-negative Breast Cancer":ti,ab OR HR-positive OR "Metastatic Breast Cancer":ti,ab OR [mh "BRCA1 Protein"] OR [mh "Genes, BRCA1"] OR [mh "Genes, BRCA2"] OR "breast cancer susceptibility":ti,ab OR "hereditary breast cancer":ti,ab) |
| **#2** | ("DNA testing":ti,ab OR "genomic testing":ti,ab OR "mutation screening":ti,ab) OR ([mh Diagnosis] OR "genomic stability":ti,ab OR "cancer risk assessment":ti,ab OR diagnosis:ti,ab OR "diagnostic assessment":ti,ab OR "early detection":ti,ab OR "early diagnosis":ti,ab OR [mh "Genetic Testing"] OR [mh "Genetic Counseling"]) |
| **#3** | ("Clinical Management":ti,ab OR [mh "Patient Care Management"] OR "treatment sequencing":ti,ab OR "patient care management":ti,ab OR "treatment planning":ti,ab OR "clinical guidelines":ti,ab OR "management guidelines":ti,ab OR "treatment guidelines":ti,ab OR "therapeutic strategies":ti,ab OR "clinical decision making":ti,ab OR "multidisciplinary care":ti,ab OR "personalized medicine":ti,ab OR "targeted therapy":ti,ab OR chemotherapy:ti,ab OR "radiation therapy":ti,ab OR "surgical options":ti,ab OR "hormonal therapy":ti,ab OR immunotherapy:ti,ab OR "palliative care":ti,ab) |
| **#4** | ([mh "Risk Assessment"] OR "risk assessment":ti,ab OR "risk analysis":ti,ab OR "risk evaluation":ti,ab OR "risk factors":ti,ab OR "cancer risk factors":ti,ab OR "breast cancer risk":ti,ab OR "predictive modeling":ti,ab OR "prognostic factors":ti,ab OR "cancer prediction models":ti,ab OR "cancer prognosis":ti,ab OR [mh "survival analysis"] OR "survival rate":ti,ab OR [mh "genetic predisposition to disease"] OR "genetic risk":ti,ab OR "family history":ti,ab OR "hereditary risk":ti,ab OR "lifestyle risk factors":ti,ab OR "environmental risk factors":ti,ab) |
| **#5** | ([mh Recurrence] OR "cancer recurrence":ti,ab OR "breast cancer recurrence":ti,ab OR "local recurrence":ti,ab OR "regional recurrence":ti,ab OR "distant recurrence":ti,ab OR relapse:ti,ab OR "cancer relapse":ti,ab OR "breast cancer relapse":ti,ab OR "recurrent breast cancer":ti,ab OR [mh "tumor recurrence, local"] OR [mh "second primary neoplasms"] OR "secondary cancer":ti,ab OR "metachronous neoplasms":ti,ab OR "second primary cancer":ti,ab) |
| **#6** | (Asia-Pacific:ti,ab OR Afghanistan:ti,ab OR Australia:ti,ab OR Bangladesh:ti,ab OR Bhutan:ti,ab OR "Burma":ti,ab OR Brunei:ti,ab OR Cambodia:ti,ab OR China:ti,ab OR "Cook Islands":ti,ab OR "Federated States of Micronesia":ti,ab OR Fiji:ti,ab OR India:ti,ab OR Indonesia:ti,ab OR "Japan":ti,ab OR Kiribati:ti,ab OR Laos:ti,ab OR Malaysia:ti,ab OR Maldives:ti,ab OR "Marshall Islands":ti,ab OR Mongolia:ti,ab OR Nepal:ti,ab OR "New Caledonia":ti,ab OR "New Zealand":ti,ab OR Niue:ti,ab OR "North Korea":ti,ab OR Pakistan:ti,ab OR Palau:ti,ab OR "Papua New Guinea":ti,ab OR Philippines:ti,ab OR Singapore:ti,ab OR "Solomon Islands":ti,ab OR "South Korea":ti,ab OR "Sri Lanka":ti,ab OR Taiwan:ti,ab OR Thailand:ti,ab OR Timor-Leste:ti,ab OR Tonga:ti,ab OR Tuvalu:ti,ab OR Vanuatu:ti,ab OR Vietnam:ti,ab) |
| **#7**  **(Diagnosis and Management)** | #1 AND #2 AND #3 AND #6 |
| **#8**  **(Risk assessment and recurrence)** | #1 AND #4 AND #5 AND #6 |
| **Embase** | |
| #1 | ('Breast Neoplasms'/exp OR 'breast cancer'/exp OR 'Breast Cancer':ti,ab OR 'Breast Carcinoma':ti,ab OR 'early breast cancer' OR 'Malignant Breast Tumor':ti,ab OR 'Invasive Breast Cancer':ti,ab OR 'Non-Invasive Breast Cancer':ti,ab OR 'Ductal Carcinoma In Situ':ti,ab OR 'Lobular Carcinoma In Situ':ti,ab OR 'Triple-Negative Breast Cancer':ti,ab OR 'human epidermal growth factor receptor 2':ti,ab OR 'HER2-negative Breast Cancer':ti,ab OR HR-positive OR 'Metastatic Breast Cancer':ti,ab OR 'BRCA1 Protein'/exp OR 'Genes, BRCA1'/exp OR 'Genes, BRCA2'/exp OR 'breast cancer susceptibility':ti,ab OR 'hereditary breast cancer':ti,ab) |
| #2 | ('DNA testing':ti,ab OR 'genomic testing':ti,ab OR 'mutation screening':ti,ab) OR (Diagnosis/exp OR 'genomic stability':ti,ab OR 'cancer risk assessment':ti,ab OR diagnosis:ti,ab OR 'diagnostic assessment':ti,ab OR 'early detection':ti,ab OR 'early diagnosis':ti,ab OR 'Genetic Testing'/exp OR 'Genetic Counseling'/exp) |
| #3 | ('Clinical Management':ti,ab OR 'Patient Care Management'/exp OR 'treatment sequencing':ti,ab OR 'patient care management':ti,ab OR 'treatment planning':ti,ab OR 'clinical guidelines':ti,ab OR 'management guidelines':ti,ab OR 'treatment guidelines':ti,ab OR 'therapeutic strategies':ti,ab OR 'clinical decision making':ti,ab OR 'multidisciplinary care':ti,ab OR 'personalized medicine':ti,ab OR 'targeted therapy':ti,ab OR chemotherapy:ti,ab OR 'radiation therapy':ti,ab OR 'surgical options':ti,ab OR 'hormonal therapy':ti,ab OR immunotherapy:ti,ab OR 'palliative care':ti,ab) |
| #4 | ('Risk Assessment'/exp OR 'risk assessment':ti,ab OR 'risk analysis':ti,ab OR 'risk evaluation':ti,ab OR 'risk factors':ti,ab OR 'cancer risk factors':ti,ab OR 'breast cancer risk':ti,ab OR 'predictive modeling':ti,ab OR 'prognostic factors':ti,ab OR 'cancer prediction models':ti,ab OR 'cancer prognosis':ti,ab OR 'survival analysis'/exp OR 'survival rate':ti,ab OR 'genetic predisposition to disease'/exp OR 'genetic risk':ti,ab OR 'family history':ti,ab OR 'hereditary risk':ti,ab OR 'lifestyle risk factors':ti,ab OR 'environmental risk factors':ti,ab) |
| #5 | (Recurrence/exp OR 'cancer recurrence':ti,ab OR 'breast cancer recurrence':ti,ab OR 'local recurrence':ti,ab OR 'regional recurrence':ti,ab OR 'distant recurrence':ti,ab OR relapse:ti,ab OR 'cancer relapse':ti,ab OR 'breast cancer relapse':ti,ab OR 'recurrent breast cancer':ti,ab OR 'tumor recurrence, local'/exp OR 'second primary neoplasms'/exp OR 'secondary cancer':ti,ab OR 'metachronous neoplasms':ti,ab OR 'second primary cancer':ti,ab) |
| #6 | (Asia-Pacific OR Afghanistan/exp OR Australia/exp OR Bangladesh/exp OR Bhutan/exp OR ‘Burma[All fields]’ OR Brunei OR Cambodia/exp OR China/exp OR ‘Cook Islands’/exp OR ‘Federated States of Micronesia’/exp OR Fiji/exp OR India/exp OR Indonesia/exp OR ‘Japan[ MeSH Terms]’ OR Kiribati/exp OR Laos/exp OR Malaysia/exp OR Maldives/exp OR ‘Marshall Islands’/exp OR Mongolia/exp OR Nepal/exp OR ‘New Caledonia’/exp OR ‘New Zealand’/exp OR Niue/exp OR ‘North Korea’ OR Pakistan/exp OR Palau/exp OR ‘Papua New Guinea’/exp OR Philippines/exp OR Singapore/exp OR ‘Solomon Islands’/exp OR ‘South Korea’/exp OR ‘Sri Lanka’/exp OR Taiwan/exp OR Thailand/exp OR Timor-Leste/exp OR Tonga/exp OR Tuvalu/exp OR Vanuatu/exp OR Vietnam/exp) |
| **#7**  **(Diagnosis and Management)** | #1 AND #2 AND #3 AND #6 |
| **#8**  **(Risk assessment and recurrence)** | #1 AND #4 AND #5 AND #6 |

**Supplementary Table S3.** Consensus statements

| **Sl. No.** | **Consensus statement** | **Level of evidence, Grade** | **Agree**  **n (%)** | **Disagree**  **n (%)** | **Abstain**  **n (%)** | **Level of consensus** |
| --- | --- | --- | --- | --- | --- | --- |
| 1 | Pretest GC and consenting should be offered before ordering a genetic test in BC patients suspected to carry hereditary BC predisposition gene mutations and/or being considered for PARPi treatment. (N=16) | 1a, High | 16 (100) | 0 | 0 | High |
| 2 | Pretest GC and consenting can be mainstreamed by trained breast surgeons and/or oncologists or other trained HCPs (including allied healthcare personnel) to reduce the burden on cancer genetics services and to ensure timely access to test results to inform surgical and therapeutic treatment decisions. (N=16) | 1a, High | 14 (87∙5) | 2 (12∙5) | 0 | High |
| 3 | In general, the ideal time for GT for *BRCA* germline pathogenic variants in *HER2–* eBC patients are at first diagnosis of cancer. (N=16) | 1a, High | 16 (100) | 0 | 0 | High |
| 4 | If available, genetic risk assessment tools (e.g. Penn, Myriad, BRCAPRO, BOADICEA, ARICA) to enrich genetic test positivity rates might be considered in low/under‑resourced healthcare systems to select patients for GT, though some patients might be missed from these tools. Healthcare practitioners may determine thresholds for parameters used in respective risk assessment tools in their respective healthcare systems according to the available resources. (N=16) | 5, Very low | 13 (81∙3) | 1 (6∙2) | 2 (12∙5) | High |
| 5 | Post-test GC should be offered to all patients whose genetic test results are positive. Patients with negative results should be considered for post-test GC as well to avoid wrong interpretation of results, especially where family history remains suggestive of an inherited disease. (N=16) | 1a, High | 14 (87∙5) | 2 (12∙5) | 0 | High |
| 6 | Patients found with likely/pathogenic variants in *BRCA* genes should optimally be referred to a cancer genetics specialist for GC and discussions around screening/prevention and predictive testing of family members. (N=16) | 1a, High | 15 (93∙8) | 1 (6∙2) | 0 | High |
| 7 | Blood relatives (at least first- and second-degree relatives of the same side of the family) of patients with likely/pathogenic variants in *BRCA* genes should be offered GC and predictive testing. (N=16) | 1a, High | 16 (100) | 0 | 0 | High |
| 8 | In general, GT for *BRCA* germline pathogenic variants should be considered for TNBC patients, irrespective of age at diagnosis or family history. (N=16) | 1a, High | 15 (93∙8) | 1 (6∙2) | 0 | High |
| 9 | In access-related related challenges, GT for *BRCA* should be offered to HR+*/HER2–* eBC patients who may be eligible for adjuvant PARPi treatment, irrespective of age at diagnosis or family history. (N=16) | 1a, High | 14 (87∙6) | 1 (6∙2) | 1 (6∙2) | High |
| 10 | If age-based selection criteria are to be used in HR+*/HER2–e*BC, GT or *BRCA* germline pathogenic variants can be limited to patients with ≤50 years of age at cancer diagnosis with an unknown or limited family history of cancer (or ≤45 years of age at cancer diagnosis with no family history of cancer for more restrictive patient selection if there are challenges to access to GC/GT). (N=16) | 5, Very low | 12 (75) | 1 (6∙2) | 3 (18∙8) | High |
| 11 | If selection criteria are to be based on family history of cancer for HR+*/HER2–*eBC patients in resource‑constrained settings, GT for *BRCA* germline pathogenic variants can be limited to patients with known likely/pathogenic variants in high-penetrance BC susceptibility genes (including *BRCA*1/2) in at least one close blood relative with BC diagnosed at 50 years or less, epithelial ovarian nonmucinous cancer at any age, or high Gleason prostate cancer diagnosed at age less than 60 years. (N=16) | 5, Very low | 11 (68∙8) | 3 (18∙7) | 2 (12∙5) | Moderate |
| 12 | If selection criteria are to be based on the risk of cancer recurrence for HR+*/HER2–*eBC patients, GT for *BRCA* germline pathogenic variants should be offered to patients who are assessed to be at high risk of cancer recurrence irrespective of age at cancer diagnosis or family history. (N=16) | 5, Very low | 12 (75) | 3 (18∙8) | 1 (6∙2) | High |
| 13 | If a likely/pathogenic germline tumour *BRCA* variants is identified, reflex testing for *gBRCA* mutation should be offered to ascertain if the mutation may be germline in nature. (N=16) | 1a, High | 16 (100) | 0 | 0 | High |
| 14 | In patients who do not opt for germline testing, tumour *BRCA* (or a gene panel containing *BRCA*) should not be used to make screening/prevention decisions for patients and predictive testing for family members. (N=16) | 5, Very low | 14 (87∙5) | 2 (12∙5) | 0 | High |
| 15 | In patients who do not opt for germline testing, tumour *BRCA*(or a gene panel containing *BRCA)* may be considered for PARPi treatment decision for metastatic BC. (N=16) | 5, Very low | 14 (87∙5) | 0 | 2 (12∙5) | High |
| 16 | In patients who do not opt for germline testing, tumour *BRCA* (or a gene panel containing *BRCA*) may be considered for PARPi treatment decision for early‑stage BC*. (N=14) | 5, Very low | 5 (35∙7) | 5 (35∙7) | 4 (28∙6) | Low |
| 17 | Testing for g*PALB2* mutations might be relevant to inform treatment decisions in high-risk *HER2–* eBC patients. (N=16) | 5, Very low | 12 (75) | 2 (12∙5) | 2 (12∙5) | High |
| 18 | If a multigene panel test is chosen for *HER2–*eBC patients for therapeutic indications, the preferred gene panel may include (but not be limited to*): BRCA1, BRCA2, PALB2,* and *TP53.* (N=16) | 5, Very low | 16 (100) | 0 | 0 | High |
| 19 | Individuals carrying likely/pathogenic germline variants in *BRCA* (+/- other hereditary genes like *TP53* and *PALB2*) might be considered for risk‑reducing surgery. (N=16) | 5, Very low | 14 (87∙6) | 1 (6∙2) | 1 (6∙2) | High |
| 20 | Healthy individuals >25 years old carrying pathogenic/likely pathogenic variants in *BRCA* genes (+/- other hereditary genes like *TP53* and *PALB2*) should undergo BC surveillance with monthly self‑examinations, twice-a–year clinical breast examinations, and yearly mammograms (or MRI between age 25*–*35 years if resources permit). (N=16) | 5, Very low | 15 (93∙8) | 1 (6∙2) | 0 | High |
| 21 | Though there is limited clinical evidence, metastatic *HER2–*BC patients whose tumours harbour somatic *BRCA* germline pathogenic variants may benefit from PARPi*. (N=15) | 5, Very low | 14 (93∙3) | 0 | 1 (6∙7) | High |
| 22 | There is robust clinical evidence to demonstrate that PARPi significantly reduces the risk of disease recurrence and provided a clinically meaningful extension of overall survival in *BRCA* germline pathogenic variants carriers with high-risk *HER2*-negative eBC across patient subgroups. (N=16) | 1b, High | 14 (87∙5) | 0 | 2 (12∙5) | High |
| 23 | Education to the patient, caregivers, and the public to enhance awareness around GT and its implications on the treatment journey (including surveillance for unaffected at‑risk healthy individuals) is a shared responsibility between healthcare providers, the pharmaceutical industry, payors, governments, and patient advocacy groups. (N=16) | 1a, High | 14 (87∙5) | 2 (12∙5) | 0 | High |
| 24 | Risk assessment for disease recurrence should be performed at diagnosis and after surgery for all *HER2*-negative eBC patients. (N=16) | 5, Very low | 15 (93∙8) | 0 | 1 (6∙2) | High |
| 25 | All eTNBC patients who have undergone upfront surgery and have ≥pT2 or ≥pN1 disease should be considered at high risk of disease recurrence. (N=16) | 1a, High | 15 (93∙8) | 0 | 1 (6∙2) | High |
| 26 | Any eTNBC patient with *BRCA* germline known likely/pathogenic variants who have undergone upfront surgery may be considered at high risk of disease recurrence, irrespective of tumour size and nodal status. (N=16) | 2b, High | 7 (43∙8) | 8 (50) | 1 (6∙2) | Low |
| 27 | Any eTNBC patient who have failed to achieve pCR after neoadjuvant treatment and surgery should be considered at high risk of recurrence, regardless of age at diagnosis, and/or family history of cancer. (N=16) | 1a, High | 15 (93∙8) | 0 | 1 (6∙2) | High |
| 28 | Any eTNBC patient with *BRCA* germline known likely/pathogenic variants who have undergone neoadjuvant treatment and surgery and have not achieved pCR status may be considered at high risk of disease recurrence*. (N=15) | 1a, High | 15 (100) | 0 | 0 | High |
| 29 | Rate the following factors on a scale from 0 to 5 based on their relevance to determine the risk of disease recurrence in HR+*/HER2*-negative eBC patients who have undergone upfront surgery (most relevant=5, least relevant=0). (N=16) | 5, Very low | **Agree** | **Disagree** | **Undecided** |  |
| a | Histological grade 3 |  | 14 (87∙5) | 0 | 2 (12∙5) | High |
| b | Histological type (nonluminal, basal) |  | 12 (75) | 0 | 4 (25) | High |
| c | ≥4 axillary nodal status involved on pathology |  | 16 (100) | 0 | 0 | High |
| d | ≥3 axillary nodal status involved on pathology |  | 15 (93∙8) | 1 (6∙2) | 0 | High |
| e | ≥2 axillary nodal status involved on pathology |  | 11 (68∙8) | 1 (6∙2) | 4 (25) | Moderate |
| f | Primary tumour size >5 cm |  | 15 (93∙8) | 1 (6∙2) | 0 | High |
| g | Primary tumour size >2 cm |  | 8 (49∙9) | 2 (12∙6) | 6 (37∙5) | Low |
| h | Ki-67 >30% |  | 13 (81∙2) | 0 | 3 (18∙8) | High |
| i | Ki-67 >20% |  | 8 (49∙9) | 3 (18∙8) | 5 (31∙3) | Low |
| j | Absence or <20% PgRs |  | 8 (49∙9) | 3 (18∙8) | 5 (31∙3) | Low |
| k | Low level of ERs (<1%) |  | 12 (75) | 1 (6∙2) | 3 (18∙8) | High |
| l | Residual cancer burden score 3 (after neoadjuvant therapy) |  | 14 (87∙6) | 1 (6∙2) | 1 (6∙2) | High |
| m | High oncotype Dx/mammaprint/prosigna/endopredict scores |  | 16 (100) | 0 | 0 | High |
| n | Intermediate oncotype Dx/mammaprint/prosigna/endopredict scores |  | 7 (43∙7) | 3 (18∙8) | 6 (37∙5) | Low |
| 30 | It is unclear whether HR+/*HER2*-negative eBC patients who have undergone neoadjuvant therapy but who did not achieve pCR may/may not be categorised as patients at high risk of disease recurrence*. (N=15) | 1a, High | 11 (73∙3) | 3 (20) | 1 (6∙7) | Moderate |
| 31 | In HR+*/HER2*-negative eBC, patient selection should be guided by the trial eligibility criteria, but the physician’s discretion to clinical judgement may be used to identify selective high-risk cases to decide on the use of adjuvant PARPi*. (N=15) | 5, Very low | 13 (86∙6) | 1 (6∙7) | 1 (6∙7) | High |
| 32 | In HR+*/HER2*-negative eBC, disease recurrence risk assessment tools (e.g. CPS+EG score, OncotypeDx, and MammaPrint etc.) could be used to support treating physicians’ clinical judgement and adjuvant treatment decisions. (N=16) | 5, Very low | 15 (93∙8) | 0 | 1 (6∙2) | High |
| 33 | Young age at cancer diagnosis is considered an independent factor to determine the risk of disease recurrence in both eTNBC and HR+*/HER2*-negative eBC. (N=16) | 1b, High | 13 (81∙3) | 0 | 3 (18∙7) | High |

BC: Breast cancer; CPS + EG: Clinical and pathologic stage and estrogen receptor status and histologic grade; eBC: Early breast cancer; ER: Estrogen receptor; GC: Genetic counselling; g*PALB-2*: Germline partner and localiser of BRCA2; GT: Genetic testing; HCP: Healthcare practitioner; *HER-2:* Human epidermal growth factor receptor 2; HR: Hormone receptor; MRI: Magnetic resonance imaging; PARPi: Poly ADP-ribose polymerase inhibitor; pCR: Pathological complete response; PgR: Progesterone receptor; TNBC: Triple-negative breast cancer; TP53: Tumour protein 53.

*Statements No. 21, 28, 30, and 31 included responses from 15 experts, and statement No. 16 included responses from 14 experts as the remaining experts voted as “not related to the field of expertise.”

Colour coding represents:

|  | High consensus |
| --- | --- |
|  | Moderate consensus |
|  | Low consensus |

**Supplementary Table S4:** Real-world survey response

| **Sl. No.** | **Consensus statements from which the questionnaire was developed** | **Question** | **Response of HCPs**  **(N)** | **Response of HCPs**  **(%)** | | **Agreement of SCMs to the statements (%)** | **Inference** |
| --- | --- | --- | --- | --- | --- | --- | --- |
|  |  | **At what point would you offer pretest GC and consenting to BC patients suspected of carrying hereditary BC predisposition?** | | | |  |  |
| a | Pretest GC and consenting should be offered before ordering a genetic test in BC patients suspected to carry hereditary BC predisposition gene mutations and/or being considered for PARPi treatment. | Before testing any patients | 73 | 54∙5 | | 100 | A significant gap observed between real-world practice and recommendations of SCMs |
| b |  | I only provide GC after testing results | 14 | 10∙4 | |  |  |
| c |  | Only to those who have a family history | 33 | 24∙6 | |  |  |
| d |  | I do not provide GC | 14 | 10∙4 | |  |  |
|  |  | **Do you agree that pretest GC can be provided by nongeneticists after receiving proper training?** | | | |  |  |
| a | Pretest GC and consenting can be mainstreamed by trained breast surgeons and/or oncologists or other trained HCPs (including allied HCPs) to reduce the burden on cancer genetics services and to ensure timely access to test results to inform surgical and therapeutic treatment decisions. | Yes | 112 | 83∙6 | | 87∙5 | Real-world practice concurs with the recommendations of SCMs |
| b |  | No | 9 | 6∙7 | |  |  |
| c |  | Not sure | 13 | 9∙7 | |  |  |
|  |  | **When do patients usually receive GT for *BRCA* germline pathogenic variants in your practice?** | | | |  |  |
| a | In general, the ideal time for GT for *BRCA* germline pathogenic variants in *HER2*-negative eBC patients is at the first diagnosis of cancer. | At diagnosis | 64 | 47∙8 | | 100 | A significant gap observed between real-world practice and recommendations of SCMs |
| b |  | During/after completing adjuvant | 21 | 15∙7 | |  |  |
| c |  | If they are eligible for PARPi | 39 | 29∙1 | |  |  |
| d |  | After surgery | 7 | 5∙2 | |  |  |
| e |  | In metastatic settings only | - | - | |  |  |
| f |  | Abstain | 3 | 2∙2 | |  |  |
|  |  | **Who do you test for *BRCA* germline pathogenic variants in your current practice? Please select all applicable answer(s).** | | | |  |  |
| a |  | I select TNBC patients aged ≤60 years | 77 | 57∙5 | |  |  |
| b | In general, GT for *BRCA* germline pathogenic variants should be considered for TNBC patients, irrespective of age at diagnosis or family history. | All TNBC patients irrespective of age at diagnosis or with a family history | 50 | 37∙3 | | 93∙3 | A significant gap observed between real-world practice and recommendations of SCMs |
| c | Post-test GC should be offered to all patients whose genetic test results are positive. Patients with negative results should be considered for post-test GC as well to avoid wrong interpretation of results, especially where family history remains suggestive of inherited disease. | All HR+*/HER2*-negative eBC patients may be eligible for adjuvant PARPi irrespective of age at diagnosis or family history | 53 | 39∙6 | | 87∙5 | A significant gap observed between real-world practice and recommendations of SCMs |
| d | Patients found with likely/pathogenic variants in *BRCA* genes should optimally be referred to a cancer genetics specialist for GC and discussions around screening/prevention and predictive testing of family members. | I select HR+ patients aged ≤50 years, who have a family history, and/or are at a high risk of recurrence | 92 | 68∙7 | | 93∙8 | Real-world practice concurs with the recommendations of SCMs |
| e |  | Abstain | 4 | 3∙0 | |  |  |
| f |  | Others | 15 | 11∙2 | |  |  |
| **5.** |  | **In my practice, for my patients with positive *BRCA* germline pathogenic variants, GC is offered to their blood relatives.** | | | |  |  |
| a | Blood relatives (at least first- and second-degree relatives of the same side of the family) of patients with likely/pathogenic mutations in *BRCA* genes should be offered GC and predictive testing. | Yes | 95 | 70∙9 | | 100 | A significant gap observed between real-world practice and recommendations of SCMs |
| b |  | No | 5 | 3∙7 | |  |  |
| c |  | If asked by the patient | 25 | 18∙7 | |  |  |
| d |  | Abstain | 9 | 6∙7 | |  |  |
|  |  | **What test would you recommend for identifying patients with *BRCA* germline pathogenic variants in *HER2-*negative eBC? Please select all applicable answer(s).** | | | |  |  |
| a |  | Germline test with *BRCA1/2* genes only | 43 | 32∙1 | |  |  |
| b | If a multigene panel test is chosen for *HER2*-negative eBC patients for therapeutic indications, the preferred gene panel may include (but not be limited to) *BRCA1* and *BRCA2*. | Germline test with multigene panel containing *BRCA1/2* | 93 | 69∙4 | | 100 | Real-world practice concurs with the recommendations of SCMs |
| c |  | Germline test for *BRCA1/2*; if negative, followed by somatic test | 6 | 4∙5 | |  |  |
| d |  | Somatic testing with *BRCA1/2* genes only | 2 | 1∙5 | |  |  |
| e |  | Somatic test with multigene panel containing *BRCA1/2* | 13 | 9∙7 | |  |  |
| f |  | Somatic test for *BRCA1/2*; if positive, followed by a germline confirmation test | 13 | 9∙7 | |  |  |
| g |  | *BRCA*: Germline to all and somatic test for patients who do not opt for germline testing | 16 | 11∙9 | |  |  |
| h |  | Abstain | 5 | 3∙7 | |  |  |
|  | Education to the patient, caregivers, and the public to enhance awareness around GT and its implications on the treatment journey (including surveillance for unaffected at-risk healthy individuals) is a shared responsibility between HCPs, the pharmaceutical industry, payors, governments, and patient advocacy groups. | **In my country, genetic testing is not well implemented due to (Please select all applicable answer[s])** | | | | 87∙5 | Real-world practice concurs with the recommendations of SCMs |
| a |  | Cost of GT | 110 | 82∙1 | |  |  |
| b |  | Cost of treatment | 89 | 66∙4 | |  |  |
| c |  | Lack of genetic counsellors leading to a long waiting time | 78 | 58∙2 | |  |  |
| d |  | Patients’ awareness | 66 | 49∙3 | |  |  |
| e |  | Lack of MDT discussions | 37 | 27∙6 | |  |  |
| f |  | Abstain | 7 | 5∙2 | |  |  |
|  |  | **When do you assess the risk of disease recurrence for your eBC patients?** | | | |  |  |
| a |  | At first diagnosis only | 12 | 9∙0 | |  |  |
| b |  | After surgery only | 6 | 4∙5 | |  |  |
| c | Risk assessment for disease recurrence should be performed at diagnosis and after surgery for all *HER2*-negative eBC patients. | At diagnosis and reassessment after surgery | 107 | 79∙9 | | 93∙8 | Real-world practice concurs with the recommendations of SCMs |
| d |  | Abstain | 9 | 6∙7 | |  |  |
| **9.** |  | **In your practice, what type of eTNBC patients do you consider at high risk of recurrence? Please select all applicable answer(s).** | | | |  |  |
| a | All eTNBC patients who have undergone upfront surgery and have ≥pT2 or ≥pN1 disease should be considered at high risk of disease recurrence. | Patients who have undergone upfront surgery and have ≥pT2 or ≥pN1 | 96 | 71∙6 | | 93∙8 | Real-world practice concurs with the recommendations of SCMs |
| b |  | Patients who have undergone upfront surgery and have *BRCA* germline likely/pathogenic variants | 61 | 45∙5 | |  |  |
| c | Any eTNBC patient who has failed to achieve pCR after neoadjuvant treatment and surgery should be considered at high risk of recurrence, regardless of age at diagnosis and/or family history of cancer. | Patients who have undergone neoadjuvant treatment and surgery but failed to achieve pCR | 103 | 76∙9 | | 93∙8 | Real-world practice concurs with the recommendations of SCMs |
| d |  | *BRCA* germline pathogenic variant carriers who have achieved pCR after undergoing neoadjuvant treatment and surgery | 22 | 16∙4 | |  |  |
| e |  | All eTNBC patients regardless of initial treatment outcomes | 41 | 30∙6 | |  |  |
| f |  | Abstain | 8 | 6∙0 | |  |  |
| **10.** |  | **What clinical factors do you evaluate to make the final decision about the high risk of recurrence in HR+*/HER2*­‑negative eBC patients who have undergone neoadjuvant therapy but not pCR.** | | | |  |  |
| a |  | No need to evaluate any other clinical factors (grade, Ki67, ER, PgR, etc.) | 18 | 13∙4 | |  |  |
| b |  | If the patient has ≥1 unfavourable clinical factor | 37 | 27∙6 | |  |  |
| c |  | If the patient has ≥2 unfavourable clinical factors | 64 | 47∙8 | |  |  |
| d |  | Abstain | 12 | 9∙0 | |  |  |
| e |  | Other | 3 | 2∙2 | |  |  |
| **11.** |  | **In my clinical practice, I use risk assessment tools, such as CPS+EG scoring.** | | | |  |  |
| a | In HR+/*HER2*-negative eBC, disease recurrence risk assessment tools (e.g. CPS + EG score, Oncotype DX^®^, and MammaPrint^®^) could be used to support treating physicians’ clinical judgement and adjuvant treatment decisions. | Never | 34 | 25∙4 | | 93∙8 | A significant gap observed between real-world practice and recommendations of SCMs |
| b |  | Rarely | 57 | 42∙5 | |  |  |
| c |  | Usually | 26 | 19∙4 | |  |  |
| d |  | Always | 4 | 3∙0 | |  |  |
| e |  | Abstain | 13 | 9∙7 | |  |  |
| **12.** |  | **When considered INDEPENDENTLY from other factors, please indicate which age threshold you currently consider to be at high risk of recurrence in both eTNBC and HR+*/HER2*-negative eBC.** | | | |  |  |
| a | Young age at cancer diagnosis is considered an independent factor in determining the risk of disease recurrence in both eTNBC and HR+/*HER2*-negative eBC. | <35 years old | 36 | 26∙9 | | 81∙3 | Real-world practice concurs with the recommendations of SCMs |
| b |  | <40 years old | 52 | 38∙8 | |  |  |
| c |  | <45 years old | 36 | 26∙9 | |  |  |
| d |  | Abstain | 10 | 7∙5 | |  |  |
| **13.** |  | **In my country, GC can be streamlined through nongeneticist HCPs.** | | | |  |  |
| a | Pretest GC and consenting can be mainstreamed by trained breast surgeons and/or oncologists or other trained HCPs (including allied HCPs) to reduce the burden on cancer genetics services and to ensure timely access to test results to inform surgical and therapeutic treatment decisions. | Yes | 65 | 48∙5 | | 87∙5 | A significant gap observed between real-world practice and recommendations of SCMs |
| b |  | No | 32 | 23∙9 | |  |  |
| c |  | I do not know | 30 | 22∙4 | |  |  |
| d |  | Abstain | 7 | 5∙2 | |  |  |
| **14.** | Rate the following factors on a scale from 0 to 5 based on their relevance to determine the risk of disease recurrence in HR+/HER2-negative eBC patients who have undergone upfront surgery (most relevant=5, least relevant=0). | **Rate the following factors on a scale of 0–5 based on their relevance to determine the risk of disease recurrence in HR+*/HER2*-negative eBC patients who have undergone upfront surgery.** | | | |  | Real-world practice concurs with the recommendations of SCMs |
|  |  |  | Disagree  N (%) | Undecided  N (%) | Agree  N (%) |  |  |
| a | Histological grade 3 | Histological grade 3 | 4 (3∙0) | 15 (11∙2) | 115 (85∙8) | 87∙5 |  |
| b | Histological type (non-luminal, basal) | Histological type (nonluminal, basal) | 10 (7∙5) | 21 (51∙7) | 103 (76∙9) | 75 |  |
| c | ≥4 axillary nodal status involved in pathology | ≥4 axillary nodal status involved in pathology | 1 (0∙7) | 5 (3∙7) | 128 (95∙5) | 100 |  |
| d | ≥3 axillary nodal status involved in pathology | ≥3 axillary nodal status involved in pathology | 4 (3∙0) | 13 (9∙7) | 117 (87∙3) | 93∙7 |  |
| e | ≥2 axillary nodal status involved in pathology | ≥2 axillary nodal status involved in pathology | 21 (15∙7) | 57 (42∙5) | 56 (41∙8) | 68∙7 |  |
| f | Primary tumour size >5 cm | Primary tumour size >5 cm | 2 (1∙5) | 17 (12∙7) | 115 (85∙8) | 93∙7 |  |
| g | Primary tumour size >2 cm | Primary tumour size >2 cm | 40 (29∙9) | 54 (40∙3) | 40 (29∙9) | 49∙9 |  |
| h | Ki-67 >30% | Ki-67 >30% | 4 (3∙0) | 30 (22∙4) | 100 (74∙6) | 81∙2 |  |
| i | Ki-67 >20% | Ki-67 >20% | 35 (26∙1) | 44 (32∙8) | 55 (41∙0) | 49∙9 |  |
| j | Absence or <20% progesterone receptors | Absence or <20% progesterone receptors | 36 (26∙9) | 57 (42∙5) | 41 (30∙6) | 49∙9 |  |
| k | Low level of oestrogen receptors (<1%) | Low level of oestrogen receptors (<1%) | 10 (7∙5) | 32 (23∙9) | 92 (68∙7) | 74∙9 |  |
| l | Residual cancer burden score 3 (post neo-adjuvant therapy) | Residual cancer burden score 3 (post neoadjuvant therapy) | 4 (3∙0) | 21 (15∙7) | 109 (81∙3) | 87∙4 |  |
| m | High Oncotype Dx/ MammaPrint/ Prosigna/ EndoPredict scores | High Oncotype Dx/MammaPrint/Prosigna/EndoPredict scores | 2 (1∙5) | 15 (11∙2) | 117 (87∙3) | 100 |  |
| n | Intermediate Oncotype Dx/ MammaPrint/ Prosigna/ EndoPredict scores | Intermediate Oncotype Dx/MammaPrint/Prosigna/EndoPredict scores | 17 (12∙7) | 59 (44) | 58 (43∙3) | 43∙7 |  |

BC: Breast cancer; CPS + EG: Pretreatment clinical and post-treatment pathological stage + estrogen-receptor status and grade; eBC: Early breast cancer; ER: Oestrogen receptor; eTNBC: Early triple-negative breast cancer; GC: Genetic counselling; GT: Genetic testing; HCP: Healthcare practitioner; *HER2+*: Human epidermal growth factor receptor 2 positive; HR+: Hormone receptor positive; MDT: Multidisciplinary team; PARPi: Poly ADP-ribose polymerase inhibitor; pCR: Pathological complete response; PgR: Progesterone receptor; SCM: Steering committee member; TNBC: Triple-negative breast cancer.

Colour coding represents:

|  | Real-world practice concurs with the recommendations of SCMs |
| --- | --- |
|  | A significant gap observed between real-world practice and recommendations of SCMs |
|  | A slight gap observed between real-world practice and recommendations of SCMs |

**Supplementary Figure Legends**

**Supplementary Figure S1.** Country-wise distribution of healthcare practitioners

**Supplementary Figure S2.** Primary speciality of healthcare practitioners
